# Development of a New Biomarker Model for Predicting Preterm Birth in Cervicovaginal Fluid

**DOI:** 10.3390/metabo12080734

**Published:** 2022-08-09

**Authors:** Ji-Youn Lee, Sumin Seo, Bohyun Shin, Se Hee Hong, Eunjin Kwon, Sunwha Park, Young Min Hur, Dong-Kyu Lee, Young Ju Kim, Sang Beom Han

**Affiliations:** 1 College of Pharmacy, Chung-Ang University, 84 Heukseok-ro, Dongjak-gu, Seoul 06974, Korea; 2 Department of Obstetrics and Gynecology and Ewha Medical Research Institute, College of Medicine, Ewha Womans University, Seoul 07984, Korea

**Keywords:** cervicovaginal fluid (CVF), preterm birth (PTB), vaginal microbiome, short chain fatty acid (SCFA), polar metabolite, biomarker model

## Abstract

Preterm birth (PTB) is a social problem that adversely affects not only the survival rate of the fetus, but also the premature babies and families, so there is an urgent need to find accurate biomarkers. We noted that among causes, eubiosis of the vaginal microbial community to dysbiosis leads to changes in metabolite composition. In this study, short chain fatty acids (SCFAs) representing dysbiosis were derivatized using (*N-tert*-butyldimethylsilyl-*N*-methyltrifluoroacetamide, MTBSTFA) and targeted analysis was conducted in extracted organic phases of cervicovaginal fluid (CVF). In residual aqueous CVF, polar metabolites produced biochemistry process were derivatized using methoxyamine and *N*,*O*-bis(trimethylsilyl)trifluoroacetamide (BSTFA), and non-targeted analysis were conducted. Nine SCFAs were quantified, and 58 polar metabolites were detected in 90 clinical samples using gas chromatography/mass spectrometry (GC/MS). The criteria of statistical analysis and detection rate of clinical sample for development of PTB biomarkers were presented, and 19 biomarkers were selected based on it, consisting of 1 SCFA, 2 organic acids, 4 amine compounds, and 12 amino acids. In addition, the model was evaluated as a suitable indicator for predicting PTB without distinction between sample collection time. We hope that the developed biomarkers based on microbiota-derived metabolites could provide useful diagnostic biomarkers for actual patients and pre-pregnancy.

## 1. Introduction

Preterm births (PTBs), which account for about 11% of all births, indicate a birth before 37 weeks, which is a cause of many problems, so it is urgently necessary to develop biomarkers that accurately predict [1]. PTB has caused several problems for premature babies. Since the main growth and development of the fetus proceeds during the last stages of pregnancy, the shorter the gestation period, the more closely monitoring needs to be for the lung, heart, and brain problems, and can exhibit behavioral problems such as attention deficit disorder and suffer from long-term health problems due to the many complications [2]. Families can suffer from social isolation, additional financial burden, and emotional instability because of the intensive care needed for premature babies. In other words, PTB is a social problem that extends beyond the level of the individual [3]. However, there is still a lack of a universal database that predicts PTB in advance, and the medical defense system is incomplete.

There are various causes of PTB. Approximately 75% are spontaneous, resulting from either spontaneous preterm labor of an undamaged membranes or spontaneous premature rupture of membranes (PROM), which may result from an infection such as chorioamnionitis that weakens the fetal membrane [4].

The remaining 25% are based on medical judgment due to factors such as maternal and fetal health problems [5,6]. Spontaneous PTB without membrane damage is caused by abnormal fetal development, chronic diseases such as maternal hypertension and diabetes, stress, and bacterial vaginosis (BV). Of them, BV is associated with dysbiosis of microbial communities, which is known to be associated with an increased risk of PTB [7,8,9]. Changes in vaginal microbial communities lead to changes in metabolite composition [10]. In a healthy vagina, *Lactobacillus crispatus* was the predominant species, and it was reported that the concentration of metabolic components such as lactic acid and pyruvic acid was high. On the other hand, a decrease of lactic acid and an increase of short chain fatty acid (SCFA) were confirmed during BV, and most amino acids were confirmed at lower levels [11,12,13,14,15]. In the vaginal mucous membrane, glucose metabolism progresses and can be affected by the microbiome, which affects the production of organic acids [16,17]. In addition to BV, inflammation of the cervix and a short cervix are known risk factors for PTB [7,18,19,20].

Traditionally, vaginal infections were diagnosed through sensory pathology and pH measurement, and the profiling of the metabolic components of CVF was mainly analyzed with nuclear magnetic resonance (NMR). Recently, papers have studied PTB by molecular phenotypes such as sialic acid, and mucin by applying a chromatography method [21,22,23]. In addition, studies have been reported that PTB can be diagnosed in early first trimester using specific miRNAs or proteins [24,25]. However, PTB caused by dysbiosis has a limit that is difficult to explain accurately with a single component. Therefore, we aimed to develop a universal biomarker that can explain the phenomenon of PTB using gas chromatography/mass spectrometry (GC/MS) and understand the metabolic process.

SCFAs, metabolites that may represent dysbiosis, were selectively extracted using non-polar solvents and the targeted analysis in CVF. In addition, polar end-point metabolites produced by biochemical processes were profiled in the residual aqueous CVF. The significant signals of metabolites were identified by comparing concentrations between groups of PTB and term birth (TB), and biomarkers for PTB were selected. Moreover, we verified the effectiveness of the selected biomarkers and the statistical method used. Finally, the metabolic pathway of the selected biomarker was presented, preparing the grounds for predicting PTB.

## 2. Results

### 2.1. Detection of SCFAs and Polar Metabolites in CVF

**Figure 1:**
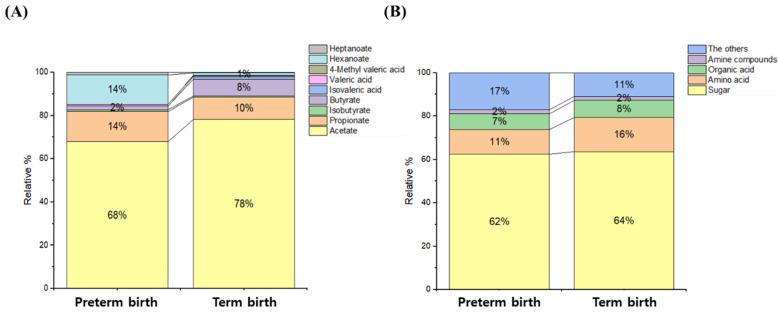
Classification of compounds (or groups) of metabolites in CVF samples. The organic acid layer of the CVF sample consisted of acetate, propionate, isobutyrate, butyrate, isovalerate, valerate, 4-methyl valerate, hexanoate, and heptanoate. The aqueous layer of the CVF sample consisted of sugar, amino acid, organic acid, amine compound, and the other group. (**A**) SCFAs of the organic layer, (**B**) polar metabolites of the water layer.

As part of the targeted analysis, quantitative and qualitative analysis were performed on 9 SCFAs, while as part of non-targeted analysis, 58 metabolites and relative quantitative were profiled. In the organic layer of CVF, the SCFAs were detected the most in descending order of acetate and propionate, while in the aqueous layer of CVF, polar compounds were detected the most in descending order of sugar, amino acid, and organic acid (Figure 1).

**Figure 2:**
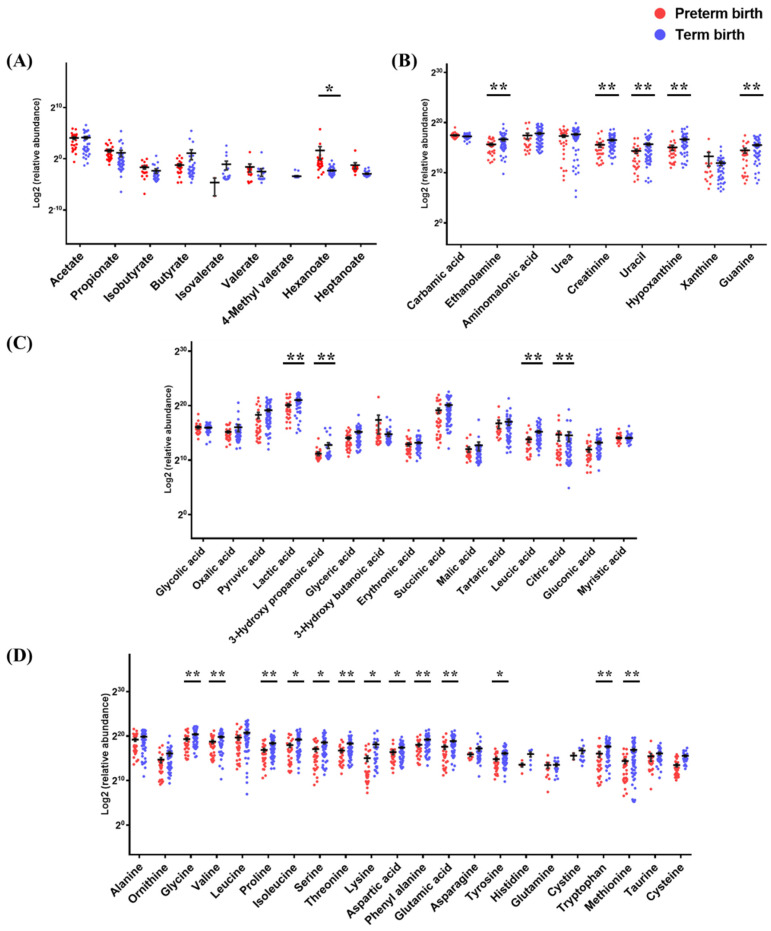
The metabolite levels of PTB and TB in cervicovaginal fluid. The results of raw data were explained as mean ± SEM. The graph used scatter dot plot of individual values and significance levels were determined by multiple *t*-test, and asterisks denote the post-test significance level (* *p*  <  0.05, ** *p* < 0.01) of PTB (20−36 weeks) and TB (37−41 weeks). (**A**) short chain fatty acids, (**B**) amine compounds, (**C**) organic acids, and (**D**) amino acids.

PTB and TB were classified based on 37 weeks gestation, and the concentration differences between the groups were compared (Figure 2 and Figure S1). In the organic layer, the concentrations of hexanoate, heptanoate, valerate, isobutyrate, and propionate were higher by 15-, 3.2-, 2.1-, 1.7-, and 1.3-fold in PTB than in TB samples, respectively, whereas butyrate was found to have a concentration 4.3-fold higher in TB than in PTB samples, and the detection rates of isovalerate and 4-methyl valerate in clinical samples were less than 30% (Table S1).

In the residual aqueous layer, 15 organic acids were detected. Compared to TB samples, PTB samples had 6.1- and 1.1-fold higher concentrations of 3-hydroxybutyric acid and citric acid, but 3.0-, 2.6-, 2.4-, and 2.2-fold lower concentrations of 3-hydroxypropanoic acid, leucic acid, gluconic acid, and glyceric acid, respectively. Twenty-two amino acids were detected. Lysine, methionine, cysteine, tryptophan, threonine, proline, serine, and ornithine were 8.4-, 5.7-, 4.2-, 3.0-, 3.0-, 2.9-, 2.8-, and 2.7-fold higher, respectively, in TB than in PTB samples. The asparagine, glutamic acid, and tyrosine concentrations were 2.5-fold higher in TB than in PTB samples. Isoleucine, phenylalanine, cystine, glycine, leucine, valine, and aspartic acid were all detected at more than 2.0-fold higher concentrations, and alanine and taurine concentrations were also higher in TB than in PTB samples. Nine amine compounds were detected. Hypoxanthine was 3.0-fold higher, and uracil, ethanolamine, and guanine were 2.5-, 2.0-, and 2.0-fold higher, respectively, in TB than in PTB samples. Six sugar metabolites were detected. The mannose concentration was 2.0-fold higher, and ribose and glucose, which had high absolute detectable levels, were 1.7- and 1.6-fold higher, respectively, in TB than in PTB samples. In addition, six metabolites not classified were detected. Comparing the concentrations of inorganic metabolites between the TB and PTB samples, sulfuric acid was 5.7-fold higher in PTB, and glyceryl phosphate and glycerol were 1.8- and 1.4-fold higher, respectively, in TB (Table S2).

### 2.2. Development of Biomarker Predicting PTB in CVF

**Figure 3:**
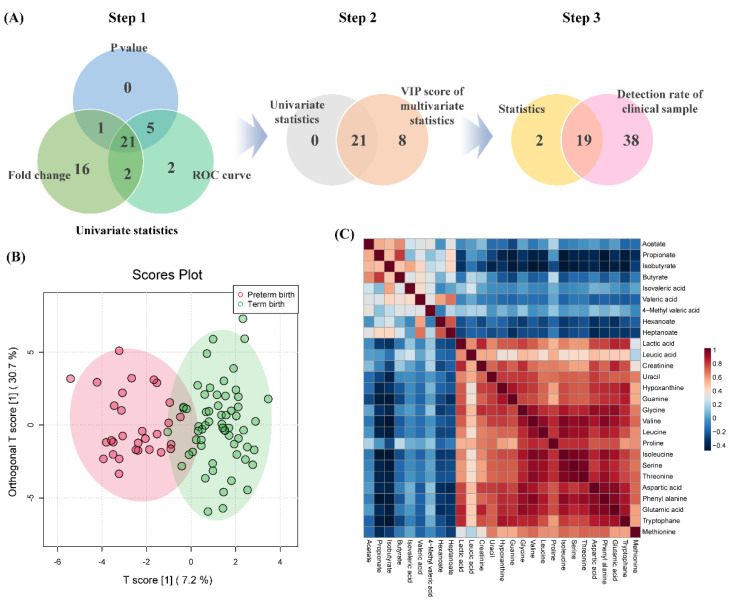
The biomarker selection process for PTB prediction and correlation between biomarker candidates and SCFAs. (**A**) Step 1; Venn diagram of *p*-value, ROC curve, and the fold change of univariate statistics processes, step 2; the overlap selected components in primary process and VIP score of multivariate statistics, step 3; a component that satisfies the detection rate of 50% or more, (**B**) OPLS−DA score plots of PTB and TB, (**C**) comparison of correlation patterns between polar metabolites of biomarker and SCFAs.

The statistical analysis of 67 metabolites detected in 90 clinical samples was performed. To select important variables for predicting PTB, *p*-values (<0.05), receiver operating characteristic (ROC) curve (≥0.7), and fold change (≥2.0) were evaluated (Table S3). Overlapping components were selected from the three evaluation indexes and used in the primary process for selecting biomarker candidates (Figure 3). In the secondary process, the intersection of the metabolites selected in the primary processes and the components satisfying the variable importance in projection (VIP) score (≥1) were selected. Finally, the components corresponding to the criteria of above a 50% detection rate in the clinical sample were selected as the final biomarkers, and arginine and 4-methyl valerate were excluded from the candidates because their detection rate was less than 50%.

The final selected biomarkers model consisted of 1 SCFA, 2 organic acids, 4 amine compounds, and 12 amino acids, and performed OPLS-DA to visualize differences between the two groups. Additionally, it was confirmed that SCFA was negatively correlated with the polar metabolite of the selected biomarker. Furthermore, predicting capability of the used model was validated using cross-validation parameters, and the results of accuracy, R2, and Q2 values were 0.86, 0.75, and 0.38, respectively (Figure S2). Permutation testing (2000 times) was performed to validate the effectiveness of the used model (*p*-value < 0.0005).

### 2.3. Evaluation of Biomarkers in Clinical Samples

**Figure 4:**
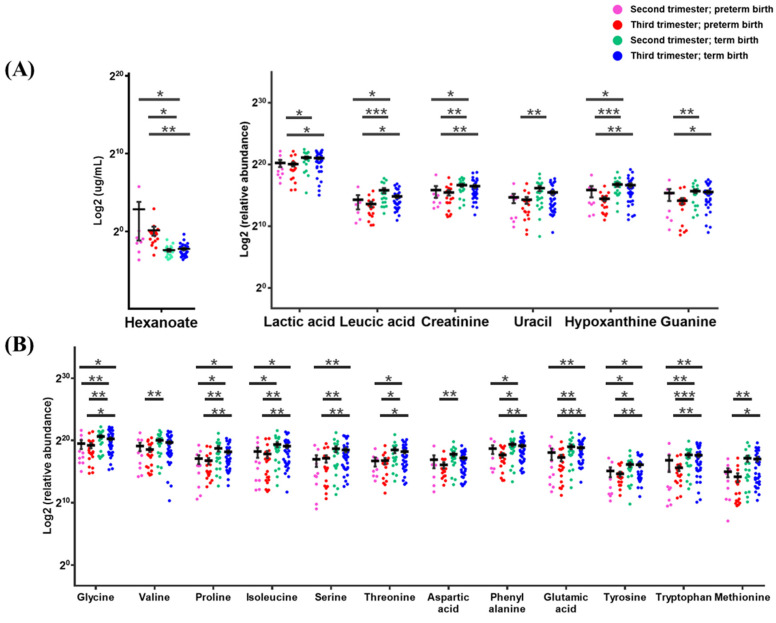
An evaluation of 19 biomarkers model for prediction PTB in CVF classified into 4 groups. (**A**) hexanoate, organic acids and amine compounds and (**B**) amino acids; CVF samples were classified into the 2nd trimester (14−28 weeks) and 3rd trimester (29−40 weeks) according to sampling week, and further classified into PTB (gestational age < 37 weeks) and TB (gestational age ≥ 37 weeks), respectively. The results of raw data were explained as a minimum to maximum of raw data. The graph used scatter dot plot of individual values and significance levels were determined by multiple *t*-test, and with asterisks denote post-test significance levels (* *p*  <  0.05, ** *p* < 0.01, *** *p*  <  0.001) between groups.

Selected biomarkers were applied to clinical samples to evaluate the effectiveness of predicting PTB. Ninety clinical samples were classified into either second or third trimester, based on the sampling weeks, and then divided into PTB and TB based on gestational age, and classified into 4 groups (pregnancy 1st trimester: 1–14 weeks, pregnancy 2nd trimester: 15–28 weeks, pregnancy 3rd trimester: 29–42 weeks). The association between the clinical samples of each group and the nineteen biomarkers were set then compared (Figure 4).

**Figure 5:**
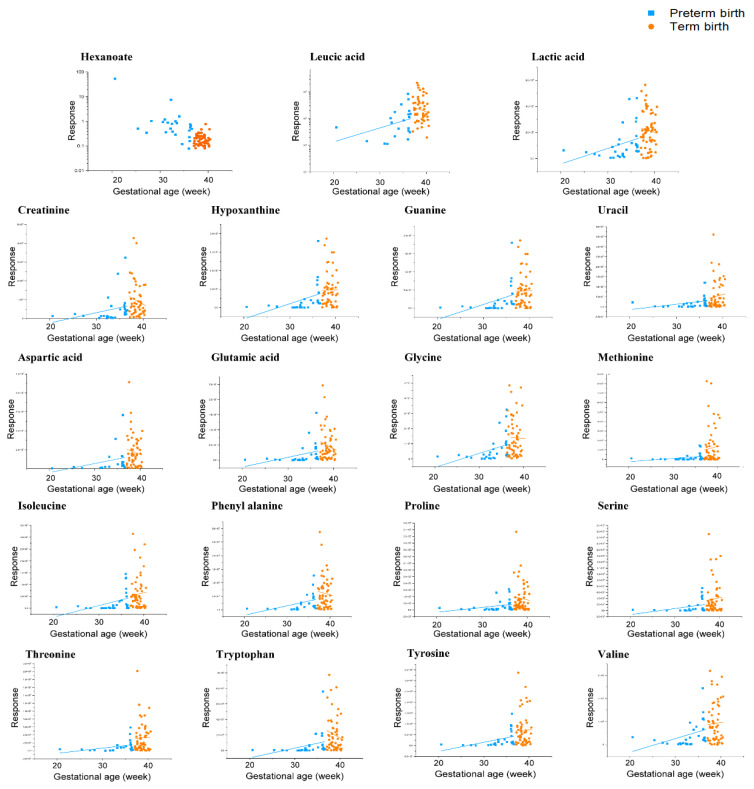
A comparison of the correlation between each of the biomarker candidates and the gestational age of CVF classified into PTB (gestational age < 37 weeks) and TB (gestational age ≥ 37 weeks). The results of the hexanoate and leucic acid were logarithmically transformed.

The biomarkers were identified to have significant signals in clinical samples (*p* < 0.05). Of the biomarker model, only the average concentration of hexanoate was higher in TB (2nd and 3rd trimester) than in PTB (2nd and 3rd trimester). The average concentrations of 18 biomarkers of polar metabolites were lower in PTB (2nd and 3rd trimester) than in TB (2nd and 3rd trimester). Methionine and threonine concentrations were detected at 5.1- and 3.0-fold higher, respectively, and proline, serine, leucic acid, uracil, and hypoxanthine were more than 2.5-fold higher in TB (2nd and 3rd trimester) than in PTB (2nd and 3rd trimester). Tyrosine, isoleucine, glutamic acid, glycine, valine, and phenylalanine were all more than 2.0-fold higher in TB (2nd and 3rd trimester) than in PTB (2nd and 3rd trimester). In addition, of the model of 19 biomarkers, 16 components were found at lower concentrations in the 3rd trimester than in the 2nd trimester of PTB, while 17 components were higher in the 2nd trimester than in the 3rd trimester of TB. As a result of comparing the biomarker concentrations of the 3rd trimester of PTB and the 2nd trimester of TB, me-thionine, hypoxanthine, leucic acid, tryptophan, uracil, and proline showed distinct con-centration differences amounting to 8.3-, 4.9-, 4.7-, 4.7-, 3.9-, and 3.9-fold, respectively. Meanwhile, glutamic acid, threonine, aspartic acid, phenylalanine, and guanine were more than 3.0-fold higher; isoleucine, serine, valine, tyrosine, and glycine were more than 2.5-fold higher; and creatinine and lactic acid were more than 2.0-fold higher in 2nd trimester of TB. In addition, leucic acid, hypoxanthine, and tryptophan in the 3rd trimester of PTB and the 2nd trimester of TB, and glutamic acid in the 3rd trimester of PTB and the 3rd trimester of TB were particularly significant (*p* < 0.001). In addition, the correlation be-tween the gestational age of clinical samples and each metabolite of the selected biomarkers model was compared, and it was confirmed that the concentration of all components was proportional to the increase in gestational age (Figure 5).

## 3. Discussion

The reason why SCFA analysis is important in this study is that SCFA is a metabolite representing the major microbiota of BV, such as *Fusobacterium* and *Prevotella* [13,17,26,27]. In PTB of CVF, the concentration of 8 SCFAs excluding butyrate, was found to be higher. On the other hand, the concentration of butyrate was found to be 4-fold higher in TB. Acquired SCFAs provide a variety of evidence for an association between the microbiome and PTB. SCFAs such as acetate and propionate, improve glucose and lipid metabolism and regulate the immune system and the inflammatory responses of the lungs and guts [28,29,30]. However, increased SCFA in a vagina is associated with bacterial growth that contributes to vaginal dysbiosis, while SCFA metabolized by carbohydrates may contribute to increase pH of the vagina [9,31]. There are contradictory reports that *Fusobacterium nucleatum*, which is observed in BV, produces abundant butyrate, and that *Firmicutes*, which has a negative correlation with the *Fusobacterium nucleatum*, also produces butyrate [32,33,34]. Butyrate has also been linked to an anti-cancer effect and increases or suppresses cancer cells depending on its amount [35,36]. In addition, *Fusobacterium nucleatum* has been reported to produce large amounts of SCFA by fermenting amino acids and other nutrient sources, a finding consistent with the results of this study [37]. In addition, no anti-inflammatory effect was observed when cervical epithelial cells were treated with a mixture of metabolic components containing a low concentration of lactic acid and high concentrations of SCFA, which is the representative phenomenon of BV, at pH 7 [38]. Human immunodeficiency virus-1 (HIV) was strongly inactivated when tested at pH 3.8 in eubiosis, but in BV, the active protonated form was decreased at pH above SCFA pKa, and HIV-1 inactivation was also low [39,40]. *Sneathia sanguinigens*, *Sneathia amnii*, *Mobiluncus mulieris*, and *Prevotella amnii* induces the production of inflammatory cytokines such as IL-1α, IL-1β, and IL-8 in cervical epithelial cells in vitro, and in vivo models, *Gardnerella vaginalis* increases the level of a pro-inflammatory cytokine such as TNF-α, IL-1β, and IL-6 [41,42]. Thus, the problem is that microbiota, which causes BV, produces not only SCFA but also inflammatory cytokines. These results have provided evidence for a correlation between PTB and SCFA, the evidence for which will become clearer if additional microbial identification studies are conducted.

The anaerobic microbiota of BV affects polar metabolites such as organic acid and amino acid. Glycogen and its breakdown products (glucose and maltose) are the main energy source for bacteria in glycolysis. During eubiosis, glycolysis steps are upregulated and lead to amino acid production and, during dysbiosis, amino acids are used as an energy source and are related to amino acid catabolism. In this study, lactic acid was detected 1.9-fold higher in TB, a finding which suggests that *Lactobacillus* predominates in this microbial environment. The vagina is a special organ that must maintain acidic conditions to prevent bacteria entering from the outside given that the lumen and the outer lumen are connected. Any increase of pH or BV formation induces inflammation, which can affect the fetus or the duration of gestational age. A strain of bacteria that can survive even at low pH is *Lactobacillus*, and its representative metabolic component is lactic acid [43]. By contrast, the bacteria of a woman during BV did not grow well below pH 4.5 and produced relatively low amounts of lactic acid [44]. The lactic acid is predominantly in the protonated form below pKa 3.9, and the anion form predominates above it. The protonated form of lactic acid is membrane-permeant, having antibacterial properties that enter the cell, acidify the cytosol and interfere with bacterial function, inducing death [40]. In particular, lactic acid has much stronger antibacterial activity than bacteria which acidy using HCl or acetic acid, which reduces the production of pro-inflammatory mediators IL-6 and IL-8 in cervical epithelial cells and induced anti-inflammatory response [45,46,47]. In addition, taurine was detected at levels 1.6-fold higher in TB. Its component affect is cholesterol degradation and lipid metabolism of the vagina and is associated with retinal dysfunction during deficiency or induction of diabetes or the inhibition of fetal brain development concentrated in the last trimester [48]. Methionine, which has a major effect on cell metabolism and various signaling steps and is also known to be associated with placental abruption and PTB, was detected in this study at 4.6-fold higher concentrations in TB [49,50,51]. The aromatic amino acid tryptophan, tyrosine, and phenylalanine, which contribute to protein structure and are used as a neurotransmitter, were detected 3.0-fold, 2.5-fold, and 2.3-fold higher in TB, respectively. Proline and ornithine were detected 2.9-fold and 2.7-fold higher, while other amino acids were also detected higher in TB. The proteolysis system of *Lactobacillus* species (*L. crispatus*, *L. jensenii*, and *L. iners*) supplies amino acids to bacteria [52]. In addition, *Lactobacillus* showed a strong positive correlation with lactic acid and amino acid such as isoleucine, leucine, tryptophan, phenylalanine, and aspartic acid, but a negative correlation with SCFA. In the BV environment, hypoxanthine, creatinine, and lactic acid were negatively correlated [10]. In addition, uracil, which affects the synthesis of polysaccharide and transport of sugars and contributes to the synthesis of cellular enzymes through binding with ribose and phosphate, was detected at levels more than 2.5-fold higher in TB. Glycerol and glyceryl phosphate, which are basic precursors of lipid metabolism, an important component in cell membrane construction, and also related to metabolism and cell signaling, were detected at levels significantly higher in TB [53]. By contrast, 3-hydroxybutyric acid was detected very significantly in PTB. It has been reported that it has been detected in ovarian serous carcinoma effusions and strongly differentiates cervical cancer patients in CVF [54,55]. Further studies extended the scope of clinical samples are needed to explain the signature of these end-point metabolites. In addition, if a study of the metabolic mechanisms of individual microorganisms related to BV is conducted, the possibility of preventing PTB as well as explaining the correlation between PTB and metabolites will be higher. The results of this study provide indirect evidence that the microbial environment affects not only SCFAs, but also polar metabolites produced by biochemical processes.

Various studies have been conducted to define the cause of PTB in the relevant literatures. Of the metabolites of *Lactobacilli*, it was found that hydrogen peroxide had the effect of reducing subsequent pro-inflammatory molecules and ascending infections of the uterus associated with chorioamnionitis and was associated with a decrease hydrogen peroxide during PTB [56,57]. Bacteria such as *Gardnerella vaginalis* or *Prevotella bivia* break down sialic acid in cervical mucus and cause epithelial cell damage. They also increase apoptosis and can lead to other viral infections [58,59,60]. Several studies have focused on the correlation between PTB and female hormones. In primates, it was confirmed that when the estrogen level was at its peak, the pH of the vagina was lowest [61]. The acidity of the female vagina rises before ovulation, and estrogen application to a near-neutral vagina after menopause increases the pH to reduce the risk of infection [62,63]. The closer the vagina of menopausal women is to neutrality, the more the reduction in glucose metabolism is associated with the absence of *Lactobacillus*. Increasing estrogen levels accelerate glycogen production in cervical epithelial cells, and the glycogen is metabolized to lactic acid by *Lactobacillus* [46,64]. In addition, it was found that injecting 17α-hydroxyprogesterone into women who had obstetric history during early pregnancy was effective in reducing the rate of PTB [65]. Many studies have reported on a relationship between cervical length and PTB. The risk of cervical shortening during the 2nd trimester of pregnancy has been associated with high levels of inflammatory cytokines IL-6 and IL-10 during early pregnancy [19]. When the length of the cervix was 15 mm or less, the risk of PTB was found to be more than 3-fold higher and associated with IL-6. In addition, CVF U. urealyticum and IL-6 and intra–amniotic inflammation was associated [66,67].

The metabolic pathway of the selected biomarkers model are mainly products of the metabolism processes of carbohydrates, amino acids, nucleotides, and energy [14,68]. The concentrations of hypoxanthine, guanine, and uracil in PTB samples of CVF were low. These are metabolites of nucleotides, which play a central role in the physiology of organisms. In the metabolic process from carbohydrates to amino acids, oxaloacetate is converted to aspartic acid, which is metabolized to lysine, methionine, threonine, and isoleucine. Aromatic amino acids (tryptophan, tyrosine, and phenylalanine) are produced from chorismate produced by oxidizing glucose in the pentose phosphate pathway. Based on these data, we can predict that the metabolism of carbohydrates and amino acids is proceeding actively. Citrate is produced by energy metabolism and metabolized to glutamic acid. Although not selected as biomarker candidates, glycerol and glycerol-3-phosphate, both produced by lipid metabolism, were detected at low concentrations in PTB. Moreover, pyruvate is a precursor of lactic acid and valine and is also associated with SCFA metabolism. Glucose is a precursor of creatinine, glycine, and serine, and glutamic acid is a precursor of proline. Thus, it can be confirmed that PTB and TB have significant differences in the metabolism of carbohydrates, amino acids, nucleotides, and energy (*p* < 0.05) (Figure S2).

**Figure 6:**
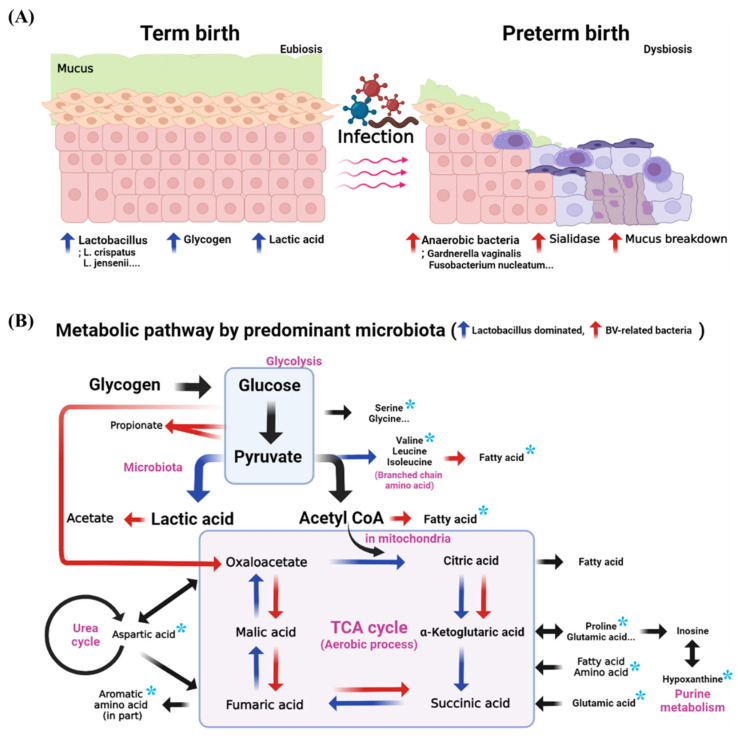
The expected metabolic pathway of PTB and TB in the vagina. (**A**) schematic representation of PTB and TB in a vagina, (**B**) comparison of the metabolic pathway between the environment of *Lactobacillus* dominance and bacterial vaginosis. The asterisks denote the selected biomarkers. Vaginal mucus acts as a chemical barrier to infection and as a primary defense in maintaining aerobiosis. The blue arrow is the metabolic direction when *Lactobacillus* dominates, and the red arrow is the metabolic direction in the BV environment. Glucose is a precursor of creatinine, glycine, and serine, while glutamic acid is a precursor of proline. Thus, it can be confirmed that PTB and TB have significant differences in the metabolism of carbohydrates, amino acids, nucleotides, and energy (*p* < 0.05).

In eubiosis, glycogen accumulates, and glycogen is metabolized by microbiota to produce amino acids; thus, the amino acid concentration may increase in TB. However, anaerobic bacteria associated with BV secrete sialidase. Sialidase breaks down mucus and produces a pore-forming substance in the vaginal epithelium, which, in turn, reduces mucus levels. Given this, amino acids would have been detected at lower concentrations in PTB than in TB (Figure 6). The production of glycogen, a major nutrient for bacteria, is accelerated by estrogen. Different members of the amylase family of enzymes have different activities depending on the type of bacteria and are involved in glycogen catabolism [69,70]. Amylase also catabolizes glycogen to maltose and maltose dextrin [71]. The produced sugars, such as maltose, are catabolized to pyruvate via glycolysis, then further catabolized by amino acid metabolism. *Lactobacillus* produces lactic acid after producing branched-chain amino acids, such as leucine, isoleucine, and valine. BV-associated bacteria, such as *Prevotella*, utilize pyruvate to generate SCFAs, as well as branched-chain fatty acids by branched-chain amino acid catabolism [27,72,73,74,75]. Therefore, the predominant microbial environment of the vagina changes metabolic pathways and affects the metabolites. In addition, reversible reactions are catalyzed by proteins and enzymes, which are associated with the bacteria of the vagina. However, the actual function of individual strains and the processes that contribute to the degradation activity are not known with any precision, so further research is needed.

Of the causes of spontaneous PTB, BV causes inflammation of the cervix and, in turn, can be transmitted from the placenta to the amniotic cavity and then to the fetus. The routes of microbial infection include from the vagina and the cervix, hematogenous transmission through the placenta, retrograde through the oviduct in the abdominal cavity, and medical processes, such as amniocentesis [76,77,78,79]. This ascending infection is a common occurrence [76,77,78,79]. In the current study, almost all (8/9) of the SCFAs were detected at higher levels, and most polar metabolites were detected at lower levels in the CVF from the PTB group compared to the TB group. When the CVF samples are compared based on the relationship between PTB and vaginal infection, SCFAs are known to be associated with *Fusobacterium*, *Prevotella*, *Gardnerella*, and BV, and amino acids are known to be negatively correlated with these bacteria. In addition, because BV is associated with PTB, it is possible to estimate the association between vaginal infections, PTB, and fetal health.

## 4. Materials and Methods

### 4.1. Sample Preparation

As the extraction process of short chain fatty acid (SCFA) in CVF, the 50 μL of sample was into a 0.5 mL microtube, and 40 μL of 2-ethyl butyrate (internal standard, 1 μg/mL) in *tert*-butyl methyl ether (MTBE) was added. The mixture was vortexed for 30 s and centrifuged (2200× *g*, 4 °C) for 5 min. The supernatant (organic layer) was transferred to another tube that contained 15 mg of MgSO_4_ and the extraction process was repeated twice using MTBE as extraction solvent. When then transferred to a 2 mL vial and inserted the collected extract. We added 10 μL of *N-tert*-butyldimethylsilyl-*N*-methyltrifluoroacetamide (MTBSTFA) with 1% *tert*-butyldimethylchlorosilane reagent into a vial, with derivatization at 80 °C for 30 min and cooled at room temperature for 20 min. The final solution was then injected into the GC/MS instrument (Figures S4 and S5).

To analyze polar metabolites in residual aqueous CVF, 50 μL of glutamic acid-*d*_5_ (internal standard, 10 μg/mL) in methanol was added into the 0.5 mL microtube. The mixture was vortexed for 30 s and N_2_ purge. Then, we added 30 μL methoxyamine in pyridine (20 mg/mL) and methoxyamination was conducted at 37 °C for 90 min. Add 30 μL of *N*,*O*-bis(trimethylsilyl)trifluoroacetamide (BSTFA) with 1% trimethylchlorosilane reagent into microtube and derivatize at 90 °C for 60 min. The derivatized final solution was cooled to room temperature and transferred to a vial and injected into the GC/MS instrument.

### 4.2. GC/MS Analytical Methods

Gas chromatography was performed on an Agilent 7890B GC system from Agilent Technologies (Santa Clara, CA, USA) and mass spectrometry was performed on an Agilent 5977A MSD from Agilent Technologies. HP-5MS UI (30 m × 0.25 mm, 0.25 μm) was used as the chromatographic column.

One microliter of derivatized SCFA was injected into GC/MS and a split ratio of 7:1 and the flow rate of helium as carrier gas was kept at 1 mL/min. The initial GC oven temperature set was 50 °C (2 min held), ramped to 150 °C at the rate of 10 °C/min, and ramped 310 °C at the rate of 15 °C/min (12.4 min held) for a total runtime of 35 min. The injector temperature, GC/MS interface, ion source, and quadrupole were 240, 300, 230, and 150 °C, respectively. The ionization was carried out in the electron impact (EI) mode at 70 eV. The MS data were acquired in selected ion monitoring (SIM) mode and the analytes were quantified using the target ion and confirmed by confirmative ions. The target ion (*m*/*z*) of acetate, propionate, isobutyrate, butyrate, isovalerate, valerate, 4-methylvalerate, hexanoate, heptanoate, and 2-ethyl butyrate (internal standard) are 117, 131, 145, 145, 159, 159, 173, 173, 189, and 173, respectively.

One microliter of polar metabolite was injected into GC/MS and split ratio of 5:1 at 280 °C of injector temperature. The initial GC oven temperature set was 50 °C (3 min held), ramped to 150 °C at the rate of 5 °C/min (2 min held), and ramped 300 °C at the rate of 2 °C/min (15 min held) for a total runtime of 65 min. The MS data were acquired in full scan mode from *m*/*z* 50 to 550. The other conditions of mass spectrometry were the same as the SCFA analysis conditions.

### 4.3. Data Processing and Statistical Analysis

Data analysis was performed using MassHunter Workstation GC/MS Data Acquisition (ver. 07.00), Agilent MassHunter Qualitative Analysis (ver. 10.0), Agilent MassHunter Quantitative Analysis (ver. 10.1), Quantitative Analysis Library Editor (Ver. 10.1) and NIST Mass Spectral Search Program (Ver. 2.3).

Each acquired data was normalized by Cube root transformation and Pareto scaling, and *t*-test and multivariate statistical analysis were performed using MetaboAnalyst (Ver. 5.0). The results of PCA, PLS-DA, and OPLS-DA were used to discover significant indicators between PTB and TB in addition to visual representation of clustering by variation of metabolite. Statistical model validation was performed to prevent overfitting of multivariate analysis using cross validation and permutation test (2000 times). The results of the data were explained as mean ± SD or SEM. ROC curve and fold change were obtained in univariate analysis, unpaired *t*-test and scatter plots were obtained using GraphPad Prism (Ver. 6.0). OriginPro 2021 (Ver. 9.8.0.200) was used for visualization of classification of relative metabolite amounts and correlation analysis of metabolite-clinical sample information. Biomarker candidates selected in statistical analysis were conducted perform pathway enrichment analysis using Kyoto Encyclopedia of Genes and Genomes (KEGG, http://www.genome.jp/kegg/ (accessed on 1 February 2022) database and MetaboAnalyst. Illustration of biochemical pathway created with BioRender.com.

### 4.4. Validation

Method validation was performed to prove reliability of the developed method. Method validation was performed on the following items: selectivity, linearity, lower limit of quantification (LLOQ), accuracy, precision, and recovery based on the ‘Guidance for Industry: Bioanalytical Method Validation’ of Food and Drug Administration (FDA) on bioanalytical method validation [80] (Figures S6–S8, Tables S4 and S5).

### 4.5. Study Population

In a nested case-control study, 90 pregnant women with PTB (*n* = 30) and TB (*n* = 60) were selected (Figure S9, Table S6). They were visited Ewha Womans University Mokdong Hospital and Hanyang University Hospital from January 2019 to March 2021 and their cervicovaginal fluid (CVF, Ewha Womans University Mokdong Hospital approval no. EUMC 2018-07-007, Hanyang University Hospital approval no. 2018-09-009) samples were collected by vaginal swabs at the second and third trimester of gestation.

## 5. Conclusions

In this study, a targeted analysis of SCFA representing dysbiosis and a profiling study of polar metabolites produced in the biochemistry process was performed using GC/MS by two methods applied to CVF. Nine SCFAs were quantified, and 15 organic acids, 22 amino acids, 9 amine compounds, 6 sugar, and 6 other components were detected in 90 clinical samples. The criteria of univariate and multivariate statistical analysis and a detection rate of clinical sample for development of PTB biomarkers were presented, and 19 biomarkers were selected based on it, consisting of 1 SCFA, 2 organic acids, 4 amine compounds, and 12 amino acids. In addition, the biomarker model was applied to clinical samples, and evaluated as a suitable indicator for predicting PTB without distinction between sample collection time within the 2nd and 3rd trimester. It is necessary to apply the developed method to more clinical samples, and further research is needed to identify a more accurate cause of vaginal microbial environment. In this study, we first developed the SCFA analysis method and a biomarker model consisting of multiple components in CVF and demonstrated the possibility that metabolic signature based on microbiota-derived metabolites can be used as a biomarker for predicting PTB. We hope that the developed biomarker model based on microbiota-derived metabolites in our study will be used as diagnostic biomarkers for actual patients and pre-pregnancy.

## Data Availability

The data presented in this study are available on request from the corresponding author. The data are not publicly available due to ethical reasons.

## Supplementary Materials

The following supporting information can be downloaded at: https://www.mdpi.com/article/10.3390/metabo12080734/s1, Figure S1: Metabolite level of preterm birth and term birth in cervicovaginal fluid, Figure S2: Validation of the statistical model used, Figure S3: Metabolic pathway, Figure S4: Comparison of derivatized-SCFA chromatograms using MTBSTFA and BSTFA reagent, Figure S5: Optimization of preparation process of short chain fatty acid, Figure S6: GC/MS chromatogram of blank sample, Figure S7: GC/MS chromatogram of blank sample spiked nine SCFAs, Figure S8: Calibration curves of nine SCFAs, Table S1: Quantitative results of SCFA in clinical sample, Table S2: Peak area of polar metabolites in clinical sample, Table S3: Results of statistical analysis, Table S4: Range of calibration curves of nine SCFAs, Table S5: Accuracy, precision, and recovery results of nine SCFAs, Figure S9: Study flowchart for subject selection criteria, Table S6: Clinical characteristics of subjects.
