# Supplementary material for: Development of a New Biomarker Model for Predicting Preterm Birth in Cervicovaginal Fluid
Source: Metabolites. 2022 Aug 9;12(8):734. doi: 10.3390/metabo12080734 (PMC9416165; doi:10.3390/metabo12080734)
Supplement: Supplementary file 1 [file metabolites-12-00734-s001.zip › metabolites-1817283-supplementary.pdf]

Contents Supplementary

Table S1. Quantitative results of SCFA in clinical sample

Table S2. Peak area of polar metabolites in clinical sample

Table S3. Results of statistical analysis

Figure S1. Metabolite level of preterm birth and term birth in cervicovaginal fluid.

Figure S2. Validation of the statistical model used

Figure S3. Metabolic pathway

Figure S4. Comparison of derivatized-SCFA chromatograms using MTBSTFA and BSTFA reagent

Figure S5. Optimization of preparation process of short chain fatty acid

Figure S6. GC/MS chromatogram of blank sample

Figure S7. GC/MS chromatogram of blank sample spiked nine SCFAs

Figure S8. Calibration curves of nine SCFAs

Table S4. Range of calibration curves of nine SCFAs

Table S5. Accuracy, precision, and recovery results of nine SCFAs

Figure S9. Study flowchart for subject selection criteria

Table S6. Clinical characteristics of subjects

Table S1. Quantitated results of SCFA analysis in clinical sample

(Unit : μg/mL)

Patient	Acetate	Propionate	Isobutyrate	Butyrate	Isovalerate	Valerate	4-Methyl valerate	Hexanoate	Heptanoate
1	N.D.	0.883	N.D.	0.143	N.D.	BLOQ	BLOQ	0.132	N.D.
2	N.D.	0.895	N.D.	N.D.	N.D.	N.D.	BLOQ	0.149	N.D.
3	N.D.	0.836	N.D.	0.047	N.D.	N.D.	BLOQ	0.164	N.D.
4	3.003	0.767	N.D.	0.065	N.D.	N.D.	BLOQ	0.162	N.D.
5	N.D.	0.819	N.D.	BLOQ	N.D.	BLOQ	BLOQ	0.136	N.D.
6	20.037	0.223	0.049	0.193	0.073	0.065	0.083	0.206	0.104
7	1.64	N.D.	N.D.	0.091	N.D.	N.D.	BLOQ	0.255	N.D.
8	N.D.	1.316	N.D.	0.108	N.D.	N.D.	BLOQ	0.219	N.D.
9	40.196	10.585	0.096	14.971	0.102	0.067	0.083	0.106	0.095
10	42.765	0.452	0.481	0.34	2.402	2.405	0.089	0.103	0.101
11	N.D.	N.D.	N.D.	N.D.	N.D.	N.D.	0.202	0.187	N.D.
12	18.737	0.256	0.056	0.208	0.067	0.065	0.083	0.082	0.086
13	0.538	0.489	0.128	N.D.	N.D.	N.D.	0.225	0.481	N.D.
14	N.D.	N.D.	N.D.	N.D.	N.D.	N.D.	N.D.	N.D.	N.D.
15	33.235	0.239	0.05	0.188	0.079	0.065	0.082	0.133	0.097
16	4.997	0.5	N.D.	0.136	N.D.	N.D.	N.D.	0.185	N.D.
17	0.404	0.177	0.048	0.085	0.061	0.065	0.082	0.121	0.098
18	2.6	0.221	1.493	0.432	5.915	0.105	N.D.	0.198	N.D.
19	42.006	0.442	0.059	0.286	0.07	0.066	0.084	0.231	0.175
20	95.002	42.284	0.172	43.828	0.132	0.141	0.082	0.112	0.094
21	13.229	0.316	N.D.	0.103	N.D.	N.D.	N.D.	N.D.	N.D.
22	18.016	2.15	N.D.	1.42	N.D.	N.D.	N.D.	N.D.	N.D.
23	19.84	0.94	0.052	0.716	0.063	0.065	0.082	0.081	0.089
24	N.D.	0.236	N.D.	N.D.	N.D.	N.D.	N.D.	0.211	N.D.
25	47.404	0.463	0.062	0.207	0.063	0.068	0.082	0.104	0.107
26	N.D.	N.D.	N.D.	N.D.	N.D.	N.D.	N.D.	0.208	N.D.
27	4.898	N.D.	N.D.	N.D.	N.D.	N.D.	N.D.	0.353	N.D.
28	17.361	N.D.	N.D.	N.D.	N.D.	N.D.	N.D.	N.D.	N.D.
29	44.25	0.591	0.094	0.223	N.D.	0.26	0.096	0.475	0.263
30	11.815	0.906	0.632	0.33	1.539	N.D.	N.D.	0.349	N.D.

Patient	Acetate	Propionate	Isobutyrate	Butyrate	Isovalerate	Valerate	4-Methyl valerate	Hexanoate	Heptanoate
31	27.207	1.107	N.D.	0.164	N.D.	N.D.	BLOQ	0.121	N.D.
32	21.537	0.897	N.D.	0.238	0.0674	0.046	BLOQ	0.289	N.D.
33	N.D.	1.595	N.D.	0.148	N.D.	BLOQ	N.D.	0.566	N.D.
34	1.871	0.86	N.D.	BLOQ	N.D.	BLOQ	N.D.	0.16	N.D.
35	N.D.	1.668	BLOQ	BLOQ	N.D.	0.311	N.D.	7.551	0.217
36	0.653	0.852	N.D.	BLOQ	N.D.	N.D.	BLOQ	0.079	N.D.
37	23.914	6.387	BLOQ	0.712	N.D.	0.225	BLOQ	1.007	0.34
38	2.617	2.717	0.097	0.292	N.D.	0.121	BLOQ	1.601	0.185
39	4.143	3.533	0.177	0.384	N.D.	0.112	BLOQ	0.512	0.178
40	N.D.	1.885	0.063	0.324	0.073	0.046	BLOQ	0.758	0.112
41	31.742	8.438	0.655	1.1	N.D.	0.167	BLOQ	0.794	0.281
42	N.D.	N.D.	N.D.	N.D.	N.D.	N.D.	BLOQ	N.D.	N.D.
43	53.543	12.777	0.989	1.548	N.D.	0.266	BLOQ	1.042	0.313
44	12.472	N.D.	0.259	0.431	N.D.	N.D.	BLOQ	0.363	N.D.
45	N.D.	0.444	0.147	0.152	N.D.	2.449	N.D.	54.362	3.055
46	N.D.	N.D.	N.D.	N.D.	N.D.	N.D.	N.D.	N.D.	N.D.
47	N.D.	N.D.	N.D.	N.D.	N.D.	N.D.	N.D.	N.D.	N.D.
48	7.061	1.968	0.095	0.601	N.D.	0.448	BLOQ	0.673	0.268
49	6.129	1.326	N.D.	0.084	N.D.	0.125	BLOQ	0.426	0.191
50	21.753	4.934	0.322	0.427	N.D.	0.204	BLOQ	0.946	0.239
51	9.961	1.581	0.153	0.171	N.D.	N.D.	N.D.	0.502	0.269
52	16.629	2.731	0.318	0.321	N.D.	N.D.	N.D.	0.348	N.D.
53	3.172	0.698	N.D.	N.D.	N.D.	N.D.	N.D.	0.23	N.D.
54	4.515	1.21	N.D.	N.D.	N.D.	N.D.	BLOQ	N.D.	0.246
55	21.768	3.062	BLOQ	0.495	N.D.	N.D.	BLOQ	0.849	0.363
56	6.5	1.312	0.161	N.D.	N.D.	N.D.	N.D.	0.512	0.231
57	25.606	3.135	0.382	0.445	N.D.	N.D.	N.D.	0.891	0.298
58	59.134	7.808	0.835	1.067	N.D.	N.D.	N.D.	1.198	0.371
59	3.637	1.063	N.D.	N.D.	N.D.	N.D.	N.D.	N.D.	N.D.
60	16.32	3.666	0.157	0.303	N.D.	N.D.	BLOQ	0.387	N.D.

ID	Acetate	Propionate	Isobutyrate	Butyrate	Isovalerate	Valerate	4-Methyl valerate	Hexanoate	Heptanoate
61	5.158	N.D.	N.D.	N.D.	N.D.	N.D.	N.D.	N.D.	N.D.
62	1.886	N.D.	N.D.	0.02	N.D.	N.D.	N.D.	0.207	N.D.
63	22.74	0.437	0.063	0.194	0.064	0.067	0.083	0.131	0.122
64	2.864	0.087	N.D.	0.029	N.D.	N.D.	N.D.	0.194	N.D.
65	44.354	0.594	0.078	0.243	0.07	0.069	0.084	0.157	0.133
66	47.477	0.492	0.066	0.2	0.088	0.067	0.083	0.249	0.103
67	3.002	1.205	N.D.	N.D.	N.D.	N.D.	N.D.	0.308	0.195
68	15.514	0.257	N.D.	N.D.	N.D.	N.D.	N.D.	0.173	N.D.
69	N.D.	0.618	N.D.	N.D.	N.D.	N.D.	N.D.	0.263	N.D.
70	40.031	0.516	0.075	0.155	0.067	0.069	0.083	0.224	0.105
71	3.926	N.D.	N.D.	N.D.	N.D.	N.D.	N.D.	0.205	N.D.
72	7.608	1.476	N.D.	N.D.	N.D.	N.D.	N.D.	0.221	N.D.
73	15.911	8.056	0.119	10.01	0.267	0.276	0.083	0.09	0.085
74	3.656	N.D.	N.D.	N.D.	N.D.	N.D.	N.D.	0.125	N.D.
75	2.992	0.717	N.D.	N.D.	N.D.	N.D.	N.D.	0.265	0.197
76	1.388	N.D.	N.D.	0.14	N.D.	0.164	N.D.	0.097	N.D.
77	3.783	1.021	0.108	0.156	N.D.	0.069	0.085	0.259	0.171
78	N.D.	N.D.	N.D.	N.D.	N.D.	N.D.	N.D.	0.101	N.D.
79	16.835	1.986	0.288	0.325	N.D.	N.D.	N.D.	0.269	N.D.
80	N.D.	0.346	N.D.	N.D.	N.D.	N.D.	N.D.	0.118	N.D.
81	N.D.	BLOQ	N.D.	N.D.	N.D.	N.D.	N.D.	0.103	N.D.
82	3.37	0.731	0.046	0.141	0.064	0.065	0.082	0.11	0.092
83	3.844	1.075	0.132	0.232	0.076	0.066	0.088	0.289	0.176
84	1.989	0.538	0.091	0.137	0.065	0.066	0.085	0.16	0.112
85	14.877	4.211	0.444	0.571	0.09	0.073	0.094	0.781	0.302
86	51.123	12.697	0.167	1.535	0.21	0.219	0.083	0.102	0.092
87	N.D.	0.547	N.D.	N.D.	N.D.	N.D.	N.D.	0.219	N.D.
88	7.338	1.787	0.181	0.229	0.073	0.068	0.084	0.342	0.15
89	0.982	0.364	0.05	0.09	0.062	0.065	0.083	0.129	0.093
90	1.178	0.34	0.073	0.109	0.063	0.066	0.083	0.142	0.105

Table S2. Peak area of polar metabolites in clinical sample

Patient	Glycolic acid	Oxalic acid	Pyruvic acid	Lactic acid	3-Hydroxy Propanoic acid	Glyceric acid	3-Hydroxy butanoic acid	Erythronic acid	Succinic acid	Malic acid	Tartaric acid	Leucic acid	Citric acid	Gluconic acid	Myristic acid	Carbamic acid	Ethanol-amine	Amino Malonic acid	Urea	Creatinine	Uracil	Hypo-xanthine	Xanthine	Guanine	Sulfuric acid	Phosphoric acid	Ethylene glycol	Propylene glycol
1	70109	12222	2863291	4559321	2099	43451	17361	8611	4187374	2601	N.D.	34073	1022	6348	23920	162869	70150	984557	244928	238526	25272	43939	N.D.	20165	N.D.	6885467	59864	36586
2	33287	14071	1810513	2770009	1138	37711	15304	3295	1264718	3112	N.D.	17701	562	2160	12982	105144	48378	451644	245901	66944	17209	4535	N.D.	22679	17957	4263914	26459	16201
3	81679	51462	150609	994534	1768	23801	12128	14268	1978628	1537	N.D.	11582	4011	8922	15142	117696	52293	118809	670	37901	28879	64951	N.D.	40518	N.D.	4690610	28941	20780
4	61074	20150	1266129	4638204	2227	65054	23212	16517	385793	910	49775	N.D.	N.D.	25034	29630	161955	124248	1064688	394262	324297	120400	315339	1233	180300	24326	6988864	46673	25685
5	46907	27826	137957	1079066	1235	9717	23496	2205	120228	2098	N.D.	10386	2210	N.D.	15075	137231	40205	104804	1335	14378	17492	21422	18766	6561	24194	4235341	32466	19771
6	62873	N.D.	617615	2835636	4064	7393	41282	4029	2252050	N.D.	949936	84722	N.D.	11629	14469	207373	110331	121261	410320	62051	34742	77991	N.D.	62212	48076	5150051	31385	22110
7	72868	N.D.	60837	228890	2482	7013	51589	5582	15562	N.D.	N.D.	N.D.	18842	459	15787	545057	4139	51017	387098	3877	2306	N.D.	3855	N.D.	55091	5262221	28722	18889
8	80832	41093	237579	674447	3375	24152	112566	3354	137556	1528	54272	4242	2550	1709	16214	279119	41600	24783	66710	5638	4951	10698	N.D.	624	114494	6166706	129018	240954
9	62014	29813	23500	199905	1312	4868	18832	3996	20412	761	26300	N.D.	3674	213	12216	191832	5934	N.D.	54493	2915	N.D.	3324	714	517	N.D.	4812683	35374	13951
10	53272	5608	146137	869584	1806	12056	N.D.	8954	2000426	1756	N.D.	19368	1220	3207	13638	118539	60813	35264	373	24084	15078	15057	N.D.	11980	N.D.	5027341	31753	29727
11	100496	120040	30348	1467677	2418	49446	3172216	45949	201947	17531	N.D.	7110	306467	2202	55647	110118	49372	36159	1982	111971	405	28753	110880	13146	2757768	5879667	47750	21536
12	106537	N.D.	184509	1730508	1951	19326	9360	7546	161930	2926	236856	14445	2144	11067	14264	157881	105013	89495	194978	32148	19880	14149	N.D.	N.D.	9965	3205093	26602	27349
13	27470	24290	15471	235861	1069	6813	86282	4224	16981	1976	29885	N.D.	30672	N.D.	13656	141667	18254	N.D.	308469	N.D.	2637	3150	N.D.	N.D.	83687	3089368	14840	9723
14	43821	31799	N.D.	61039	1351	3849	N.D.	2630	13764	N.D.	N.D.	N.D.	1676	N.D.	13723	157553	4765	N.D.	26026	N.D.	306	4037	N.D.	623	N.D.	6218172	31696	93688
15	360508	14355	146254	623263	16329	23380	33272	10067	141756	26136	N.D.	4706	33319	1871	17160	178231	16587	46927	13713	13944	22244	14178	1101	1917	36528	4205822	2431046	92430
16	53934	47616	35388	1099992	1225	21660	7419	7702	81684	4243	132158	1684	6354	3600	18940	184880	152099	37172	229790	72515	55844	91855	3507	74108	N.D.	4174167	27749	21609
17	75351	94905	40265	1031475	1100	22528	8307	15596	82022	2864	71961	4393	2613	1890	8534	139080	134379	48317	168359	51162	42135	45427	1170	19317	N.D.	4253875	34088	11919
18	36476	21525	92998	541360	1428	2629	N.D.	1446	430323	N.D.	32682	3085	N.D.	538	21103	167023	45072	14892	231403	23679	3579	8799	220	11766	N.D.	4789088	66944	30569
19	156741	91240	1009207	3105032	5292	31585	17552	6305	2826621	1650	39485	53014	N.D.	5966	13416	197578	122059	208448	7067	82720	120519	30142	N.D.	82595	N.D.	8427918	370170	487608
20	30347	17710	9429	58977	1216	2857	11487	1611	5088	N.D.	13177	N.D.	1306	N.D.	8924	182837	14366	N.D.	86040	3186	2059	2049	N.D.	379	11119	3229076	17367	15782
21	27725	41313	61578	567598	1458	7248	10907	8196	120398	N.D.	52405	15053	N.D.	1305	18317	173015	66317	N.D.	321778	44506	4982	8332	2224	11329	N.D.	4508519	23677	27402
22	45140	53960	19218	333942	1564	5978	N.D.	3346	26665	1201	N.D.	1442	2830	1203	16000	224770	19285	N.D.	153567	14853	927	11779	N.D.	2732	N.D.	5786028	51556	36499
23	31545	15435	78398	576600	1339	16961	N.D.	2288	451988	N.D.	54327	3599	N.D.	1333	11003	122578	22563	23718	58444	21354	7438	29635	576	15787	N.D.	2858045	19412	9326
24	57159	36898	43131	1190328	1426	22723	N.D.	8132	67272	2838	81848	8616	1799	1928	11988	193950	84298	54532	152632	49168	5381	27411	423	12591	N.D.	6740034	31550	13620
25	68951	23276	N.D.	129783	2914	3657	47202	2668	25412	1781	N.D.	N.D.	8397	N.D.	19167	226976	6495	N.D.	321994	3732	661	N.D.	N.D.	N.D.	46755	8779303	422597	51816
26	50860	22945	36286	481512	903	7396	N.D.	4206	415287	N.D.	N.D.	N.D.	563	1046	10935	162369	38263	20646	42036	24473	2444	26742	112	5576	N.D.	3927105	20531	18948
27	29116	35732	47164	524208	975	9254	21751	7004	14924	2137	38490	1136	6220	826	11994	166637	50139	N.D.	147050	14460	11243	19889	323	1045	N.D.	4227365	56103	10468
28	58660	96921	11287	889340	1857	16300	29330	21075	133682	6220	N.D.	1168	168054	1106	33354	172128	19515	N.D.	629922	13925	N.D.	4104	3779	234	294818	4633175	22319	29699
29	14948	7501	9731	368478	N.D.	1620	N.D.	929	40059	N.D.	16077	2176	N.D.	218	6290	139246	10983	13012	1364	7518	N.D.	N.D.	N.D.	N.D.	N.D.	1806962	18783	8423
30	50441	17283	16366	125622	1387	4315	7948	2319	9426	2194	18634	N.D.	1282	N.D.	31096	261049	15760	N.D.	80969	8925	1693	3596	N.D.	677	N.D.	3625508	45101	41210
31	64698	14897	170653	3498681	1500	51253	7175	6941	202513	4382	90996	12875	8916	33556	13679	80724	184806	233802	316315	80797	111625	143983	7858	149396	N.D.	5980467	72500	133852
32	52388	9740	344774	2345414	1284	3311	N.D.	4737	1446438	782	20941	29458	N.D.	6650	9127	57522	N.D.	59543	32193	25930	3526	N.D.	N.D.	1045	N.D.	5696572	62515	647571
33	67706	28017	1355263	2717626	N.D.	81132	8147	12629	517820	1647	19831	88856	515	10700	7425	71890	56370	78551	209857	37764	22160	3037	N.D.	16786	N.D.	6074718	44511	39366
34	85348	23692	1436357	2676881	1821	323204	29182	6376	3096030	5718	5523	99792	845	54020	15675	163404	61757	225330	6232	72179	143968	220256	240	121360	28448	4985515	72809	47474
35	108935	41908	303224	2401121	2255	32012	11266	12491	78336	3247	13048	44345	6091	4183	14063	118525	32115	347391	228326	34753	35630	68663	2769	38798	11306	5599406	37867	27159
36	114757	21905	1602049	3966028	2607	65089	13201	10872	3391024	2645	5634	7978	587	8457	10211	124718	71144	222212	375173	42916	6893	37007	1868	22510	15512	5852120	43011	32552
37	93071	45675	220442	3442014	1865	80060	71756	23799	288721	3907	206770	38147	2017	19484	15944	139136	107989	599217	441359	64035	212533	303848	38099	149322	71909	5920311	48812	30904
38	85505	35611	265735	1298053	2267	41805	12910	7234	309617	3357	18985	11432	12375	5950	12945	100163	32136	60069	2814	22642	4944	11684	N.D.	4950	12628	6757536	54157	37565
39	113246	N.D.	1037146	472789	5224	63265	18647	7284	3501699	6250	10477	35672	3837	274	6392	118258	124488	297095	1950	38560	41649	3433	N.D.	242	22444	4632229	57427	53091
40	127744	26072	544622	2239124	2477	20962	N.D.	5028	92538	4416	16913	14143	14171	14806	11712	108768	N.D.	N.D.	1325	8228	N.D.	N.D.	N.D.	3607	N.D.	5847186	61339	97046
41	59673	22149	552522	2028052	1661	33359	13878	5177	368682	2117	17821	17101	626	12852	13947	127991	79238	176328	87071	42893	9398	39051	782	37532	N.D.	5085215	39869	16998
42	48146	4474	1058682	3644334	1213	17592	N.D.	6842	2521558	1851	2652	24596	529	4702	16537	133009	25867	580519	318843	104647	2182	55771	3357	10023	N.D.	4896275	36597	20672
43	78622	53561	175433	1078783	2012	13880	9677	10837	267055	2951	16549	9007	2378	12467	11929	95313	21131	58663	90923	19105	6865	5296	N.D.	3504	N.D.	6189937	55857	35336
44	68444	19982	73189	2177388	1724	34144	N.D.	12374	104077	4681	14190	4242	4352	8272	18579	123944	10581	473005	84074	64526	52312	93625	1279	97865	N.D.	5207163	35825	18261
45	59553	22361	2201444	4016516	1778	45795	N.D.	13243	3794832	6740	30426	38715	595	14641	18884	174386	77051	801963	351878	179822	22490	154941	551	63934	N.D.	5727188	35252	30270

Patient	Glycerol	Glycerol-3-phosphate	Alanine	Ornithine	Glycine	Valine	Leucine	Proline	Isoleucine	Serine	Threonine	Lysine	Aspartic acid	Phenyl alanine	Glutamic acid	Asparagine	Tyrosine	Histidine	Glutamine	Cystine	Trypto-phan	Methionine	Taurine	Cysteine	Ribose	Arabinose	Fructose	Mannose	Galactose	Glucose
1	566477	178094	1443361	59555	2386122	782220	961453	108921	285669	55282	86183	2646	315010	559335	905272	N.D.	28866	N.D.	N.D.	N.D.	215260	2775	N.D.	31694	1135646	N.D.	30053	13657	3322	458078
2	645730	148468	674767	19381	1009072	308189	398018	34249	120457	49150	74445	651	124858	271571	397726	N.D.	27313	N.D.	N.D.	N.D.	68618	824	5284	17223	612161	N.D.	14851	8732	3940	444131
3	612729	34433	199307	7648	314137	267251	339731	64088	99088	18540	20397	2040	70838	146106	105274	N.D.	13971	N.D.	N.D.	N.D.	38050	26364	15319	1752	842476	N.D.	N.D.	12060	477	354935
4	302639	379865	1239486	173641	3248876	1088294	1354586	216086	503695	78025	139935	15357	566177	1269986	1564743	154194	147096	N.D.	N.D.	72044	760269	137036	34939	43113	2445383	N.D.	N.D.	29099	13980	1410617
5	381530	28096	132454	3578	273211	216751	115782	24765	24537	6265	15454	2318	19297	84495	10428	N.D.	7141	N.D.	N.D.	N.D.	28591	10865	2670	2091	128169	N.D.	N.D.	5318	1401	231462
6	325051	74538	3152782	243483	1856890	2448396	6848573	623492	1455066	611250	424360	303551	129773	929647	559246	39915	94249	10322	52416	27290	221662	65910	492854	7527	1475133	191984	31225	9411	1252	656449
7	486454	12622	1485674	15161	646159	959017	1088956	520308	99718	156210	145457	116981	20600	209659	60860	24555	16994	3090	6286	N.D.	56444	39711	5541	7480	20524	77131	4813	2533	490	57022
8	54039	20037	1081731	22210	349859	650211	745563	247337	134089	46188	80435	4243	N.D.	102679	40554	N.D.	7215	N.D.	1790	N.D.	11347	9253	13976	2190	159880	N.D.	2346	6777	647	67447
9	158143	7600	161313	706	54178	51944	66395	32156	33594	11779	8987	923	N.D.	12295	5248	N.D.	N.D.	N.D.	N.D.	N.D.	1740	1277	6170	2954	N.D.	N.D.	N.D.	1920	N.D.	40924
10	358202	145467	192084	3284	170991	192674	121387	21131	17825	6593	11139	1985	40333	100796	51550	N.D.	10296	N.D.	N.D.	N.D.	21026	2953	N.D.	1656	453233	N.D.	N.D.	8225	14815	262633
11	646213	26569	12955	1079	443347	35035	14196	9353	12937	N.D.	N.D.	4081	N.D.	15807	20410	N.D.	10410	N.D.	N.D.	N.D.	9455	95	N.D.	44972	144556	201051	41490	20795	13356	533057
12	540129	10870	1504491	24727	1164326	1132651	2066077	315610	827877	389891	168217	101039	67296	470519	382681	71970	44591	12749	17092	N.D.	N.D.	41142	N.D.	1653	278891	66088	7630	8670	609	204237
13	81675	2997	246373	882	86254	49394	27061	64817	12244	8195	19243	711	N.D.	N.D.	7801	N.D.	1250	N.D.	1337	N.D.	806	1511	N.D.	1083	34446	N.D.	5364	4826	N.D.	60767
14	159658	13066	20651	1199	29011	23790	179136	N.D.	4227	1613	N.D.	1595	7167	N.D.	2332	N.D.	N.D.	N.D.	N.D.	N.D.	462	1544	N.D.	3205	32381	N.D.	1704	N.D.	19	9187
15	5092	101916	202892	10956	164303	325888	168277	61857	46583	26844	41233	1624	3432	47701	17675	N.D.	5543	N.D.	175	N.D.	6130	11492	4503	60333	316970	N.D.	3273048	263209	27625	214536
16	46449	113510	950728	11331	1041255	1124075	4234141	192654	1266503	456060	268968	234570	132655	595961	399165	74322	77010	23865	6834	N.D.	190496	147975	84742	15974	742114	159665	8532	12459	679	640660
17	169411	91371	954668	11451	931982	676470	1180353	104064	658551	496327	208355	132510	45117	429574	206836	33981	65913	10024	16169	N.D.	74747	63862	51843	20112	548176	90139	6196	12856	868	500074
18	2002	3335	233027	14737	258314	312578	521167	70044	135965	35570	81226	329	33114	152118	92845	N.D.	18551	N.D.	N.D.	N.D.	11942	271	12614	5430	120428	N.D.	N.D.	3915	294	282986
19	86005	353355	2338603	82571	2808353	1410715	3209870	552453	1296051	743028	589234	58702	129794	498562	436231	28425	90198	N.D.	15418	N.D.	67757	735	40156	7267	1643843	34723	16830	45648	7083	909998
20	340291	3574	37306	886	26971	22009	9784	6040	3561	N.D.	2993	782	N.D.	N.D.	N.D.	N.D.	N.D.	N.D.	N.D.	N.D.	532	988	N.D.	2394	N.D.	N.D.	N.D.	N.D.	168	5250
21	20333	23837	105335	4095	225896	159283	72498	12244	17234	5135	23755	1520	15406	60619	40918	N.D.	7204	N.D.	N.D.	N.D.	19235	11348	21372	2617	125107	N.D.	N.D.	11922	1016	294811
22	6966	78980	106954	4224	145900	82708	225756	5800	12244	N.D.	11558	1941	6812	30549	23557	N.D.	2186	N.D.	N.D.	N.D.	4490	5142	276	3845	39810	N.D.	N.D.	3965	740	53476
23	390358	99136	235887	22518	254345	203814	365789	33198	174498	42776	52409	799	28373	133677	63528	N.D.	15295	N.D.	2486	N.D.	23293	8075	24557	1787	239784	N.D.	N.D.	5187	289	222482
24	107657	116749	628103	10573	738893	382366	627377	65920	336827	186441	115062	8883	46324	238498	191313	N.D.	16974	N.D.	5386	N.D.	40396	16819	37627	5479	220459	N.D.	5189	11139	N.D.	53948
25	N.D.	9553	87555	N.D.	48590	27443	23439	27044	9455	2784	N.D.	3065	N.D.	N.D.	4964	N.D.	N.D.	N.D.	N.D.	N.D.	N.D.	948	N.D.	7471	97586	N.D.	128007	10634	3216	5785
26	64649	104135	221505	7844	261601	197012	147571	27917	86796	20351	34098	592	17896	45098	53381	N.D.	2733	N.D.	N.D.	N.D.	5381	1419	21314	2470	74482	N.D.	N.D.	3809	403	58986
27	79671	6334	64929	3766	138200	68493	49283	15559	12358	N.D.	7614	153	11872	27660	36219	N.D.	4322	N.D.	N.D.	N.D.	6792	10707	16349	1790	101615	N.D.	2811	17837	N.D.	228214
28	47496	16804	197687	6635	296155	57521	13235	33425	4444	N.D.	N.D.	1737	N.D.	12520	6506	N.D.	2318	N.D.	N.D.	N.D.	2035	1204	4942	17166	30988	N.D.	16466	25492	13286	101873
29	43200	56324	78579	563	87859	20988	11300	2226	4479	874	N.D.	905	N.D.	N.D.	5793	N.D.	N.D.	N.D.	N.D.	N.D.	1369	134	N.D.	2633	49983	N.D.	N.D.	1817	32	9073
30	125108	13009	25534	1103	33462	18765	6076	1531	4221	518	N.D.	4295	N.D.	N.D.	3502	N.D.	2816	N.D.	N.D.	N.D.	706	N.D.	N.D.	14838	25910	N.D.	N.D.	159491	144725	178621
31	320499	145602	1468433	5936	1698629	1675248	1687147	116230	723758	467576	463460	161681	296358	886713	802234	164092	202742	N.D.	N.D.	N.D.	533853	331676	48639	53751	1506526	96045	9828	141647	8007	903413
32	284939	333766	19586	2677	151687	93697	98675	6837	57736	8002	7905	N.D.	27864	98502	87292	N.D.	14216	N.D.	N.D.	N.D.	68567	1329	N.D.	N.D.	903403	52042	N.D.	16709	N.D.	752578
33	374312	27702	349945	1548	282697	256691	247305	31139	92219	51574	57398	N.D.	31020	131386	167944	N.D.	33452	N.D.	N.D.	N.D.	18404	310	N.D.	N.D.	772958	25406	9551	15278	3095	480433
34	395306	145835	1763501	205263	1449095	1869595	2730534	476251	1005376	736050	626417	33184	222594	1486400	692337	74306	220319	N.D.	4226	N.D.	451982	3418	62050	43920	1769072	N.D.	54072	345567	2486	1271704
35	650233	22830	1049054	8766	2020812	924127	1052679	78318	426785	164254	189640	30650	200051	798597	388998	134120	97399	N.D.	N.D.	N.D.	291980	204963	N.D.	25017	744864	18654	31706	13819	923	710842
36	1079909	36433	1090026	7387	1045350	981422	1634904	204107	548706	285196	328314	25617	161884	505167	470341	46692	86829	N.D.	N.D.	N.D.	173280	5252	46269	25752	503052	20169	24961	44474	15476	944783
37	456251	152557	2270744	229542	3680268	2604391	420384	893041	2144354	1694719	891900	662291	311358	1643044	995800	306735	270914	123994	6711	N.D.	815031	480513	167047	64444	1930003	432947	121282	453628	73743	1321903
38	636648	23962	70994	N.D.	266453	88405	97031	13428	48029	11321	11952	N.D.	18330	93903	45290	1979	18380	N.D.	N.D.	N.D.	39400	13559	N.D.	N.D.	328323	32815	17069	43724	182	587351
39	391648	N.D.	276784	N.D.	503767	71295	110979	204771	18598	17964	9249	N.D.	27916	36897	10126	27222	N.D.	N.D.	N.D.	43511	1093	19335	N.D.	N.D.	406453	16789	65860	11547	3376	31736
40	228258	16994	18472	N.D.	44911	6620	1739	N.D.	N.D.	N.D.	N.D.	N.D.	N.D.	27972	N.D.	N.D.	N.D.	N.D.	N.D.	N.D.	3032	50	N.D.	N.D.	900833	N.D.	21456	27934	12063	67475
41	346820	94963	283819	3404	569903	314139	277242	30107	77332	55374	38921	N.D.	68654	352284	208783	N.D.	55892	N.D.	N.D.	N.D.	138140	15470	3928	9783	441186	N.D.	10553	26406	1301	817094
42	326909	68182	1301972	111925	2132143	983067	1892951	235613	492612	313687	356148	9259	304319	762529	1007305	38067	92234	N.D.	N.D.	22345	384970	23102	21990	26051	616602	N.D.	30899	17184	4999	273247
43	525698	27574	13288	N.D.	199659	50746	34427	6581	18467	2541	N.D.	N.D.	15656	37861	35734	N.D.	7393	N.D.	N.D.	N.D.	23001	12237	N.D.	N.D.	203794	N.D.	7583	24483	1094	445484
44	412827	49310	350623	30181	1699754	726150	683244	104695	314741	62642	105766	5271	203671	795630	555939	86518	107021	N.D.	N.D.	N.D.	373482	170256	N.D.	31719	888825	N.D.	N.D.	24793	7184	306074
45	285447	195046	2227498	67409	2843979	1829089	6479221	248174	1304270	1184991	861131	83839	380685	1237269	1435560	125434	190544	N.D.	N.D.	N.D.	285263	3168	29016	111022	923099	N.D.	18493	268169	8160	631679

Patient	Glycolic acid	Oxalic acid	Pyruvic acid	Lactic acid	3-Hydroxy-propanoic acid	Glyceric acid	3-Hydroxy-Butanoic acid	Erythronic acid	Succinic acid	Malic acid	Tartaric acid	Leucic acid	Citric acid	Gluconic acid	Myristic acid	Carbamic acid	Ethanol-amine	Amino-malonic acid	Urea	Creatinine	Uracil	Hypo-xanthine	Xanthine	Guanine	Sulfuric acid	Phosphoric acid	Ethylene glycol	Propylene glycol
46	111511	69983	176855	2212254	2273	51418	N.D.	23468	268565	4395	13837	N.D.	8708	49224	16995	134000	31323	323196	141639	197852	124993	224431	4160	123516	N.D.	6024831	37591	31672
47	61660	20545	2935149	4836578	1803	68429	31022	23562	3442594	3982	15001	64579	630	27517	18251	160287	109269	910446	547774	211399	49184	440616	554	122418	31968	6013092	30507	14852
48	78169	10045	699301	3828378	2643	65546	22056	11491	789967	20377	8326	143980	12226	1149	18698	156252	40648	45681	2428	48484	26965	42032	429	14807	22295	5965108	47760	29109
49	69607	20301	2202306	4079651	2258	61337	12479	8210	270820	1401	2868	53171	751	6022	17278	149950	66452	153114	260355	83518	13669	84155	997	49558	N.D.	5779096	46200	25393
50	81337	N.D.	652591	2170084	39450	84090	37797	3864	3178319	4186	59984	117766	N.D.	N.D.	15416	180772	864402	86166	450455	401228	12096	N.D.	N.D.	N.D.	38418	5805177	99507	110045
51	56763	11434	397154	3964033	2608	27382	24383	10467	358378	N.D.	18371	5365	599	3301	20079	165992	174667	370797	388018	179933	137805	159242	1632	67212	N.D.	6311305	43137	18757
52	72259	13293	179540	2438534	N.D.	16843	N.D.	5261	2253448	1409	101525	8757	1557	8784	14979	195128	33923	38733	196379	126429	10206	42507	375	34637	N.D.	6419940	47876	36307
53	65216	19730	56706	2587416	1748	17393	39992	8819	3221270	3984	14796	73113	13194	6500	14604	138273	239975	302227	856	47858	98393	74854	117	41219	40701	4809441	49974	24374
54	91300	34213	27184	2191550	1571	42424	9162	12782	203496	728	17744	1971	N.D.	1147	14314	151680	43087	192686	119434	31508	22343	34621	425	33398	N.D.	6252444	47972	26109
55	54389	10169	2334477	5647343	2081	106104	11153	13042	301321	N.D.	25719	15120	30	6879	19089	127459	77191	538320	269622	214353	93455	234307	1250	57021	N.D.	5764946	45355	21702
56	55065	9862	1920659	5168358	1856	17960	13290	20256	4019701	6649	11499	37622	680	4340	25091	124466	30912	885995	306555	244558	9949	219044	N.D.	32204	16610	6708500	45517	20608
57	122862	N.D.	281317	1960426	25824	11653	51878	13431	6227489	175093	4708	218552	5351	1526	29711	208407	344501	620071	148454	105884	360730	106393	N.D.	6262	47883	9251345	74235	160025
58	N.D.	98914	N.D.	42975	N.D.	N.D.	N.D.	#VALUE!	175630	N.D.	152190	N.D.	24830	N.D.	17083	186971	N.D.	18471	88	N.D.	321	N.D.	N.D.	N.D.	N.D.	4970	533902	N.D.
59	7957	513548	4064	54415	N.D.	N.D.	N.D.	#VALUE!	832367	N.D.	763441	N.D.	163997	N.D.	30341	161521	N.D.	92825	37	N.D.	328	N.D.	N.D.	N.D.	N.D.	3457	1749826	7270
60	10427	1543483	8391	92447	N.D.	N.D.	246375	#VALUE!	2325127	809	2683209	N.D.	658569	N.D.	79284	241450	881	262148	1060	N.D.	273	N.D.	N.D.	N.D.	256345	3286	2392568	8695
61	34926	16329	228943	2151233	N.D.	5486	N.D.	4793	153503	718	192960	21113	2097	3495	12083	144104	143930	45060	351894	60098	12958	41954	228	39167	N.D.	4821822	33283	13927
62	65129	43562	99290	1703515	N.D.	25420	9644	7877	80694	3116	103577	13871	9330	3747	16531	164181	117698	37557	270956	65768	40271	77289	4780	53804	N.D.	5489619	37704	33026
63	66540	11118	413866	3725743	N.D.	16329	9745	7280	3701088	985	56806	15388	N.D.	5679	11547	137961	214947	133222	391673	172403	42017	100221	273	52190	N.D.	6603893	43382	20093
64	71020	35631	688968	2307643	N.D.	8718	N.D.	5300	1229127	766	73570	25142	2939	2490	11272	145757	60333	57803	170009	77788	7690	16062	106	19672	N.D.	6739215	48276	27887
65	62360	13758	909298	4465124	2006	73213	20017	19941	551736	N.D.	120591	15013	516	15335	16309	191121	460719	459946	504687	428500	179338	589133	20828	186653	21681	6215142	42536	18587
66	45288	33546	1759416	4178906	1646	31465	18868	7477	3217023	908	108517	33473	1062	4691	10048	205729	7983	199287	478140	81746	5866	49877	7979	15588	N.D.	5585962	33678	14360
67	41681	24117	279750	1374973	1190	7511	16517	2590	642187	1273	62084	14857	1881	1690	13813	156072	31313	23875	107509	22923	3647	16155	146	11406	14741	5110829	38063	13667
68	55136	27157	429967	2197066	N.D.	29702	15638	14067	1031856	5613	352255	22258	6350	26662	18087	164677	115190	107845	5690	89504	42928	134127	8169	2733	N.D.	3348316	27752	21339
69	40923	31564	287915	1828040	1457	9562	7766	3518	1063713	1011	60412	39088	675	1172	10474	141081	54193	113116	173815	21660	19375	34165	483	35800	N.D.	4064442	38963	17143
70	65181	16775	681183	3594276	1802	70759	22463	15367	415276	818	281347	22573	1583	1559	16364	206112	40345	685110	252177	240278	219684	439897	3854	168809	22473	4491830	69341	47088
71	43989	23984	214974	1266991	N.D.	10511	N.D.	3076	613141	608	10349	7924	N.D.	N.D.	10814	137880	71347	119198	250577	47453	16512	9843	216	53217	N.D.	4501592	46304	31110
72	27154	24200	111828	710348	N.D.	3637	N.D.	2480	239195	782	40352	4755	761	N.D.	14932	161429	32724	38609	108327	14840	3755	3656	N.D.	15230	N.D.	3559056	34354	22436
73	37344	4793	1617115	504525	62519	13702	N.D.	7145	716469	2215	14070	131042	1032	N.D.	17363	170525	54845	405709	131389	123556	134557	104138	81	61322	N.D.	4080938	52405	93459
74	54801	22653	164497	1362806	1405	11281	7694	4409	130070	N.D.	74647	14700	709	N.D.	13507	106782	31328	37656	99386	56076	106656	40809	N.D.	54311	N.D.	3482954	34086	78626
75	70789	90262	30872	802764	2382	10843	10439	5284	50820	2718	42200	8282	2905	3593	12010	133193	80549	15274	112355	28304	11221	42994	1296	11592	N.D.	5024254	87966	148717
76	36884	19921	138601	3365051	N.D.	57506	22851	12366	187893	6553	311734	11839	16084	16518	15027	144304	225357	747124	459628	179090	152697	183683	7732	100980	24047	4609832	23803	18073
77	55852	22807	16412	425319	N.D.	2985	N.D.	931	79524	N.D.	12918	N.D.	681	N.D.	16163	187755	28910	25238	22634	10672	N.D.	N.D.	N.D.	N.D.	N.D.	4992774	34062	35156
78	37186	19459	73443	1606178	N.D.	19031	10449	5548	79975	5685	137654	9517	6760	4793	17614	171583	25836	158583	318868	60508	24717	83938	5766	40607	N.D.	3629062	22628	19419
79	55852	22463	142999	213906	N.D.	5250	N.D.	2052	48197	539	20574	13184	679	N.D.	18518	223904	5820	N.D.	24963	3599	501	N.D.	N.D.	N.D.	N.D.	4961805	30519	12970
80	45222	19287	104324	1615791	N.D.	14851	9754	4515	144810	N.D.	187864	7135	N.D.	1179	14351	166343	95167	99086	128613	78820	33436	87531	615	42866	N.D.	3665152	23981	16866
81	77322	23451	310140	2074430	1777	18412	20584	12570	572206	5984	544241	40591	18290	9538	19664	192433	248288	213197	476514	126201	44918	144618	11062	86340	52540	4949793	33207	25142
82	110025	15559	910862	2033796	60081	80759	22261	9344	918034	5733	275337	178886	34861	1777	22256	186071	85246	355745	277868	145326	130316	33113	6325	66722	22010	7474940	43376	26452
83	101030	41337	23932	147092	N.D.	7088	14137	5212	32656	1620	17786	N.D.	7811	N.D.	21621	117942	9456	19834	121507	9200	N.D.	2117	N.D.	383	N.D.	5123641	33453	22442
84	34436	30735	17904	295821	N.D.	8813	N.D.	3894	31677	N.D.	44033	N.D.	N.D.	N.D.	13641	182642	23617	33356	90338	7813	8431	13221	N.D.	9923	N.D.	3725577	22483	29860
85	71180	186335	118680	1434936	3090	42041	172465	44247	423033	6845	N.D.	8184	158526	4392	40101	213513	75945	101842	977245	118745	5067	27744	2710	506	172234	6904254	33475	97042
86	48541	N.D.	700951	1647952	7685	18628	8598	1768	2742748	1567	N.D.	65216	725	N.D.	12128	157468	278252	158905	159018	138921	20676	17380	N.D.	901	N.D.	3709008	25015	29492
87	60900	46657	28259	1397950	N.D.	38843	N.D.	8255	77598	3868	113516	N.D.	3998	1317	17035	246962	115897	232669	339477	91727	61553	112717	2139	50652	N.D.	4160528	21683	11475
88	41031	20514	N.D.	33163	N.D.	2659	N.D.	1363	4526	N.D.	22036	N.D.	N.D.	N.D.	10239	261143	4787	16681	12697	N.D.	N.D.	N.D.	N.D.	N.D.	N.D.	4323012	33487	44314
89	25623	40813	79744	818597	N.D.	6208	N.D.	8187	1447546	2302	N.D.	3306	N.D.	2588	15617	185439	50208	65734	113751	39022	3194	71574	1206	20316	N.D.	3554780	21805	18092
90	41705	87213	70437	468671	N.D.	15164	7282	6233	37653	820	95597	6682	2516	2044	25957	222971	52829	32368	167803	14594	5576	41772	N.D.	8135	N.D.	3935828	19517	10717

Patient	Glycerol	Glycerol-3-phosphate	Alanine	Ornithine	Glycine	Valine	Leucine	Proline	Isoleucine	Serine	Threonine	Lysine	Aspartic acid	Phenyl alanine	Glutamic acid	Asparagine	Tyrosine	Histidine	Glutamine	Cystine	Tryptophan	Methionine	Taurine	Cysteine	Ribose	Arabinose	Fructose	Mannose	Galactose	Glucose
46	427767	124413	228816	11748	1673392	270259	301344	44089	91276	47781	64331	N.D.	138220	257016	303169	N.D.	52546	N.D.	N.D.	N.D.	154078	38553	6331	N.D.	1779248	N.D.	101128	100372	54294	1258526
47	270474	278084	2428170	65814	2889976	1800349	638652	335325	1102578	1328083	895077	142964	326017	1442545	1353555	117312	209053	N.D.	N.D.	N.D.	597195	6254	48358	109500	2180222	107683	53315	112904	7623	1048859
48	436719	17683	111369	7981	313309	243803	186956	44679	36431	6637	11239	N.D.	68263	249165	71671	N.D.	6536	N.D.	N.D.	N.D.	61145	16568	1576	N.D.	513463	N.D.	8756	4886	3070	48039
49	298629	86425	1547978	43371	1055227	769632	1245036	137004	342503	56985	106326	N.D.	197197	872084	579065	33476	79475	N.D.	N.D.	N.D.	223738	3403	23194	25672	857778	N.D.	35956	13303	6581	680153
50	336309	57809	1012889	0	547209	237232	193719	1330751	36871	6242	17282	N.D.	146521	84521	415254	69463	N.D.	N.D.	N.D.	164170	9936	24713	N.D.	19770	254156	23084	11758	2981	6620	29910
51	340037	136372	1588238	18233	3522241	2087987	2834283	480564	865088	347866	487245	N.D.	397897	1151309	679555	73631	83464	N.D.	N.D.	N.D.	172414	438122	N.D.	N.D.	945031	N.D.	11732	14136	8138	1082201
52	342204	141546	1653076	6940	1532299	1731521	6593370	638642	1397611	947294	554526	642905	149902	1118519	722312	84852	70320	N.D.	N.D.	N.D.	206158	159777	57639	N.D.	493401	479837	21422	16441	3390	513879
53	401176	34571	1380077	5258	1399792	834080	1260864	226265	407514	474590	366419	19683	272458	676348	460915	102249	24514	N.D.	N.D.	N.D.	246852	165706	N.D.	N.D.	718660	N.D.	58385	23997	24420	563183
54	428505	906190	545943	15961	856808	405582	267432	27319	128899	42492	34417	N.D.	156844	294719	283520	N.D.	17290	N.D.	N.D.	19353	76106	85471	N.D.	N.D.	555162	77771	N.D.	31758	1289	328694
55	341140	131828	1417216	98637	2904284	1275068	1504713	150055	488436	73434	74081	N.D.	417791	1263530	1139698	99756	36200	N.D.	N.D.	N.D.	83808	40350	N.D.	32713	452490	N.D.	18973	8046	1191	196025
56	360418	195154	1252285	117376	3195585	1024458	1500419	130359	482480	46444	74195	N.D.	345817	800015	1148088	30385	60557	N.D.	N.D.	N.D.	682651	7832	N.D.	66444	1162623	N.D.	44942	20755	4207	484662
57	746280	79219	1480340	37518	2936010	2615674	1916203	294880	567770	356105	573821	N.D.	495830	833953	214927	N.D.	46229	N.D.	N.D.	N.D.	387499	564542	N.D.	31832	1785888	N.D.	39860	18362	148718	532739
58	1393	N.D.	15075	N.D.	144822	56605	32865	17021	44532	4889	21696	N.D.	7613	28296	5473	20448	N.D.	N.D.	N.D.	N.D.	N.D.	19077	N.D.	N.D.	112542	N.D.	42160	37319	25666	463
59	1125	10281	45408	N.D.	416658	204946	176186	103210	233404	48419	156351	N.D.	83077	304356	49186	52135	N.D.	N.D.	N.D.	N.D.	N.D.	257090	N.D.	78876	282015	N.D.	150393	143944	113835	4490
60	260	9756	240953	7728	1141409	605489	541432	326436	737828	259361	663524	N.D.	243139	994192	228524	142734	13808	N.D.	N.D.	10159	N.D.	802806	N.D.	166609	628898	N.D.	355585	311494	254969	10407
61	187117	68847	1872602	156845	1619353	1415008	4724487	379953	1037363	598741	451911	516546	157088	760659	540460	75569	86587	5538	21733	N.D.	200416	155465	118336	N.D.	216206	321755	17137	18176	N.D.	423095
62	409897	220693	628697	12027	785901	737971	1571776	141768	573469	183539	167629	31492	113352	524234	318773	N.D.	72964	N.D.	5290	N.D.	187593	132248	77499	N.D.	705518	N.D.	6741	15691	1977	707384
63	394014	130895	1544553	107514	2164744	1082968	2279644	371277	835534	509745	346453	303828	260426	942060	962110	92877	63259	N.D.	6899	23451	274778	49155	32759	N.D.	999869	183871	9426	7005	105	583379
64	362191	49723	990243	37056	861455	494070	1314319	239070	342443	120169	171645	4566	43440	238001	283587	N.D.	16087	11552	N.D.	N.D.	47591	9731	45276	N.D.	460743	N.D.	9533	6633	N.D.	121407
65	218361	324599	2506803	379151	4412483	2751993	10543823	1005223	2463727	1685717	1162905	1643447	589987	2399719	2078763	932178	181995	N.D.	N.D.	87178	779512	353118	74710	105435	767537	1047372	26126	53055	1981	733943
66	693724	199692	2104683	179131	2284958	1547407	2709083	459035	818232	206501	394364	184785	156432	880755	760635	31961	69607	N.D.	19502	556655	214584	5188	86216	26120	594331	125670	20014	12795	4670	533168
67	214478	21267	364718	3466	239563	176174	329791	38180	84460	22023	56997	2054	8384	113078	75071	N.D.	12562	N.D.	N.D.	N.D.	35360	9694	30525	N.D.	336615	146139	N.D.	7356	70	338270
68	154732	53068	1675113	8231	1274613	1328436	4355048	536260	929724	491335	384568	185992	215386	725059	436909	90288	105558	N.D.	35843	26141	150644	1117	236266	10499	3308431	121783	N.D.	93512	1819	809766
69	212838	28921	741241	7315	692597	765894	769519	85922	315053	252830	242501	N.D.	87262	458815	197566	N.D.	42243	N.D.	N.D.	233501	137821	78816	32042	N.D.	286440	N.D.	N.D.	8884	2252	416287
70	247264	337916	2813047	1075996	4839596	3199085	12531792	2336875	3141873	2512406	2014138	2347035	913771	2867933	2466563	1568826	336587	107373	5748	147781	974085	826167	160195	57881	768950	1521409	17524	38427	36865	484577
71	225303	22769	355080	36450	584745	988547	1476107	301873	491431	364831	299416	56039	91172	537635	320002	19845	63139	N.D.	14435	N.D.	107334	16478	22361	N.D.	397195	32333	5602	11532	N.D.	296488
72	155557	N.D.	173285	12002	221703	247605	347576	54873	68701	85750	100204	N.D.	22971	193370	85830	N.D.	31771	N.D.	N.D.	N.D.	37622	15629	12360	N.D.	374705	N.D.	N.D.	7959	N.D.	391064
73	143917	125499	574570	15193	3055729	564348	964941	775389	293220	304439	153378	N.D.	142194	323566	303320	N.D.	20568	N.D.	N.D.	46457	11015	82573	22885	N.D.	1782120	N.D.	49218	9936	56637	194327
74	440251	132400	1826459	6683	1916384	2044756	5966728	835962	1779771	742311	441057	61918	198211	532029	352494	82525	18659	N.D.	6572	N.D.	65061	138971	31136	N.D.	613848	36307	9587	4182	636	148270
75	4115	N.D.	362823	8495	639252	730779	770750	79867	325685	143374	95494	15303	35131	269069	101978	N.D.	25455	N.D.	N.D.	N.D.	95592	71524	18148	N.D.	142364	N.D.	N.D.	10047	877	439398
76	45367	95873	2731683	104845	4700132	2943067	9133027	702720	2697087	1800958	1085039	318430	318315	972372	1034610	458788	206468	106658	20169	N.D.	473982	472375	217127	26281	1176101	190669	40652	26978	3639	1335680
77	73622	N.D.	32079	2258	172822	26878	14693	N.D.	6922	N.D.	N.D.	N.D.	N.D.	10595	13157	N.D.	883	N.D.	N.D.	N.D.	4851	42	N.D.	N.D.	12113	N.D.	2012	N.D.	N.D.	4165
78	265808	32839	598675	14320	832645	759851	1396359	149556	481496	183962	162713	N.D.	104387	506197	273160	18416	60596	N.D.	N.D.	N.D.	237112	189615	79268	21955	401857	N.D.	4560	69271	7036	581849
79	351432	N.D.	15830	N.D.	71400	1283	125	N.D.	N.D.	N.D.	N.D.	N.D.	10284	N.D.	N.D.	N.D.	N.D.	N.D.	N.D.	N.D.	N.D.	54	N.D.	N.D.	81986	N.D.	3536	3338	23	16838
80	367546	33652	636708	99330	912147	791161	1311958	165933	292989	167517	199291	7417	110649	407661	314529	24077	12095	N.D.	1184	N.D.	73930	93974	106888	N.D.	347172	N.D.	4256	N.D.	N.D.	89494
81	535617	42424	2126590	58193	1307385	1984173	2927166	795114	1507597	692640	604247	394264	309894	955307	647655	182972	116423	24498	35022	N.D.	354346	380659	347799	54986	498349	266675	89004	38311	11909	629598
82	827419	206949	1977044	53890	2581369	1030763	1447573	1148419	424112	63055	122473	N.D.	139979	415486	212395	10416	40300	N.D.	2109	N.D.	62539	139638	160145	12685	1396882	N.D.	41032	25120	406	715512
83	598848	10731	7597	1421	196893	9876	3306	N.D.	3370	N.D.	N.D.	3388	N.D.	N.D.	N.D.	N.D.	2034	N.D.	N.D.	N.D.	1445	2528	N.D.	6373	16188	N.D.	4931	5940	504	126119
84	45177	414367	134853	9493	183351	180639	209431	20327	117538	22079	25910	N.D.	13390	59900	39859	N.D.	5108	N.D.	N.D.	N.D.	8014	1921	9103	N.D.	80760	34900	N.D.	8304	29	74728
85	80698	18265	143717	679	757083	197772	95842	45139	26794	N.D.	N.D.	N.D.	6775	30652	49649	N.D.	10869	N.D.	N.D.	N.D.	38128	39	N.D.	N.D.	1065271	N.D.	14588	25375	3745	540915
86	98663	9567	1187977	5610	736196	281890	339945	1121291	99451	162657	115885	N.D.	54031	46185	266961	N.D.	5385	N.D.	N.D.	43322	9934	535	25391	N.D.	508739	N.D.	69433	17336	30227	286503
87	47074	912095	561515	90769	1040975	776481	1053081	103291	516759	211485	202659	8952	122104	356212	352122	39298	23397	N.D.	1345	N.D.	95847	93094	68204	N.D.	791875	55502	2574	46501	552	256523
88	265372	N.D.	1977	N.D.	40645	N.D.	753	N.D.	N.D.	N.D.	N.D.	N.D.	8209	N.D.	N.D.	N.D.	N.D.	N.D.	N.D.	N.D.	N.D.	39	N.D.	N.D.	45965	N.D.	N.D.	N.D.	22	3505
89	29386	191906	402822	6913	422744	278734	282248	35835	103223	42061	70516	N.D.	40070	167490	116902	N.D.	15821	N.D.	N.D.	N.D.	49315	10758	38238	N.D.	325782	12360	N.D.	34742	N.D.	397343
90	38771	126014	582351	7622	286274	337835	592951	71431	172605	67758	46386	N.D.	17135	106551	75732	15872	5477	N.D.	4054	N.D.	19405	26767	20666	N.D.	68526	15279	1715	8487	378	31884

Table S3. Results of statistical analysis

(VIP) score (≥ 1)		p values (<0.05)		Fold change (≥ 2)		ROC curve (≥ 0.7)	
Conponent	VIP score	Conponent	P value	Conponent	Fold Change	Conponent	AUC curve
Aminomalonic acid	1.5882	Aminomalonic acid	0.00027	2-hydroxybutanedionic acid	2.0514	Aminomalonic acid	0.7827
Asparagine	1.3347	Asparagine	0.00196	3-Hydroxybutanoic acid	7.8559	Asparagine	0.7034
Aspartic acid	1.8093	Aspartic acid	0.00004	3-Hydroxypropanoic acid	2.0551	Aspartic acid	0.7883
Creatinine	1.2716	Creatinine	0.00546	Arabinose	3.3981	Creatinine	0.7605
Glutamic acid	1.4898	Glutamic acid	0.00080	Asparagine	6.4675	Glucose	0.7228
Glycerol	1.0190	Glycine	0.00063	Aspartic acid	2.8172	Glutamic acid	0.7642
Glycine	1.4846	Guanine	0.00744	Citric acid	2.9518	Glyceric acid	0.7148
Guanine	1.0623	Hypoxanthine	0.00773	Creatinine	2.0583	Glycine	0.7531
Hypoxanthine	1.0166	Isoleucine	0.00367	Cystine	5.1549	Guanine	0.7327
Isoleucine	1.3027	Lactic acid	0.00095	Ethylene glycol	3.2484	Hypoxanthine	0.7460
Lactic acid	1.5146	Leucic acid	0.00070	Fructose	5.2426	Isoleucine	0.7235
Leucic acid	1.5797	Methionine	0.00089	Gluconic acid	2.4661	Lactic acid	0.7840
Leucine	1.0658	Phenyl alanine	0.00017	Glutamic acid	2.5349	Leucic acid	0.7744
Methionine	1.4767	Proline	0.00759	Glyceric acid	2.2284	Leucine	0.7074
Phenyl alanine	1.5778	Pyruvic acid	0.00716	Glycine	2.2317	Mannose	0.7111
Proline	1.2765	Ribose	0.00030	Guanine	2.2453	Methionine	0.7358
Pyruvic acid	1.2923	Serine	0.00295	Histidine	2.9128	Phenyl alanine	0.7698
Ribose	1.6368	Succinic acid	0.00866	Hypoxanthine	3.0589	Proline	0.7198
Serine	1.3398	Threonine	0.00135	Isoleucine	2.3077	Pyruvic acid	0.7611
Succinic acid	1.3448	Tryptophane	0.00049	Lactic acid	2.0300	Ribose	0.7648
Threonine	1.4197	Tyrosine	0.00424	Leucic acid	3.4193	Serine	0.7318
Tryptophane	1.3664	Uracil	0.01364	Leucine	2.2403	Succinic acid	0.7148
Tyrosine	1.1720	Valine	0.00677	Lysine	4.1900	Threonine	0.7309
Uracil	1.2282	Propionate	0.00642	Methionine	5.3313	Tryptophane	0.7963
Valine	1.1676	Heptanoate	0.00232	Ornithine	2.5081	Tyrosine	0.7392
Hexanoate	1.6420	Hexanoate	0.00057	Phenyl alanine	2.7990	Uracil	0.7185
4-Methyl valeric acid	1.6098	4-Methyl valeric acid	0.00009	Proline	2.8346	Valine	0.7241
Heptanoate	1.2426	-	-	Serine	3.1664	Propionate	0.7478
Isovaleric acid	1.1072	-	-	Sulfuric acid	8.6995	Hexanoate	0.7456
-	-	-	-	Threonine	3.3205	4-Methyl valeric acid	0.7250
-	-	-	-	Tryptophane	3.1904	-	-
-	-	-	-	Tyrosine	2.7747	-	-
-	-	-	-	Uracil	2.7906	-	-
-	-	-	-	Valine	2.1534	-	-
-	-	-	-	Hexanoate	13.5490	-	-
-	-	-	-	Heptanoate	3.5964	-	-
-	-	-	-	Isovaleric acid	12.7100	-	-
-	-	-	-	Butyrate	4.1664	-	-
-	-	-	-	4-Methyl valeric acid	3.1293	-	-

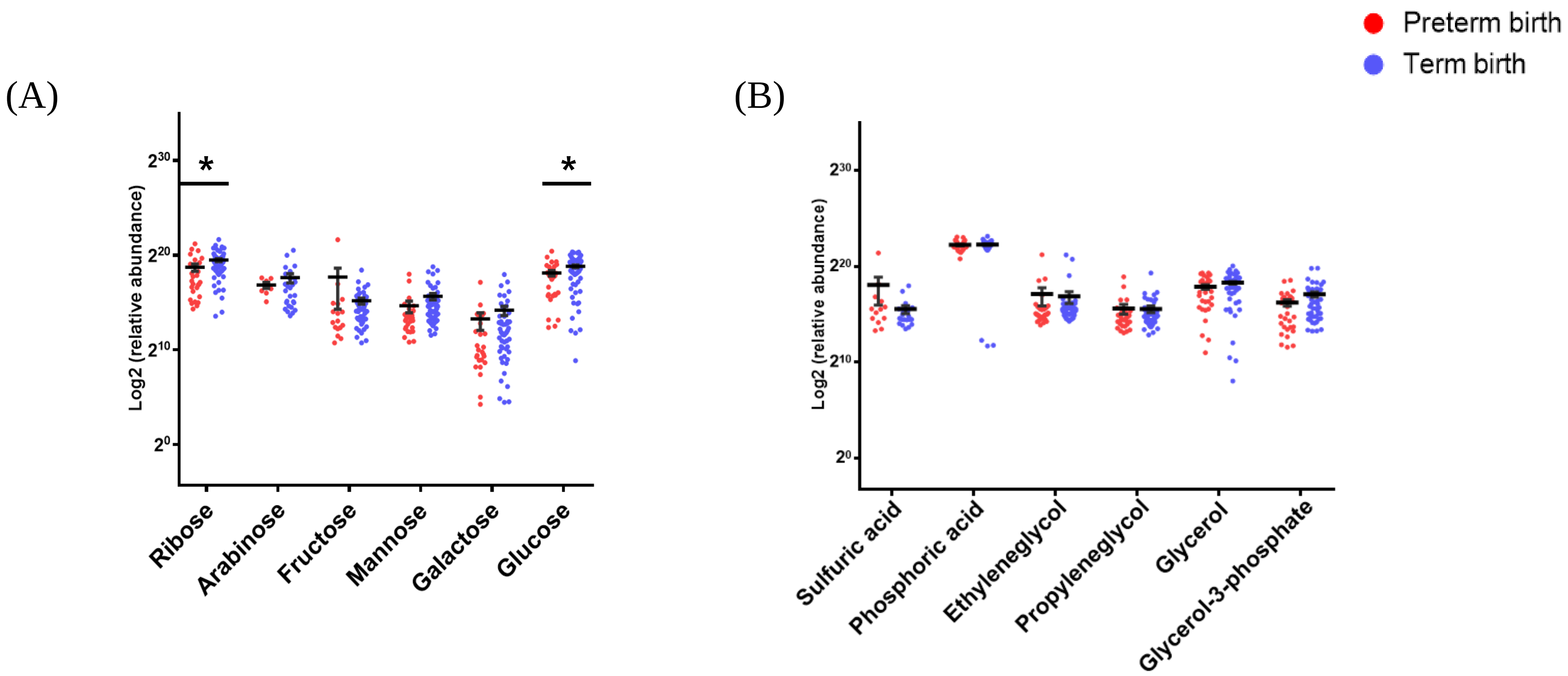

Figure S1. Metabolite level of preterm birth and term birth in cervicovaginal fluid.

The results of raw data were explained as mean ± SEM. The graph used scatter dot plot of individual values and significance levels were determined by multiple t-test, and asterisks denote the post-test significance level (*$p < 0.05$) of preterm birth (20-36 weeks) and term birth (37-41 weeks). (A) sugar and (B) the others

(A)

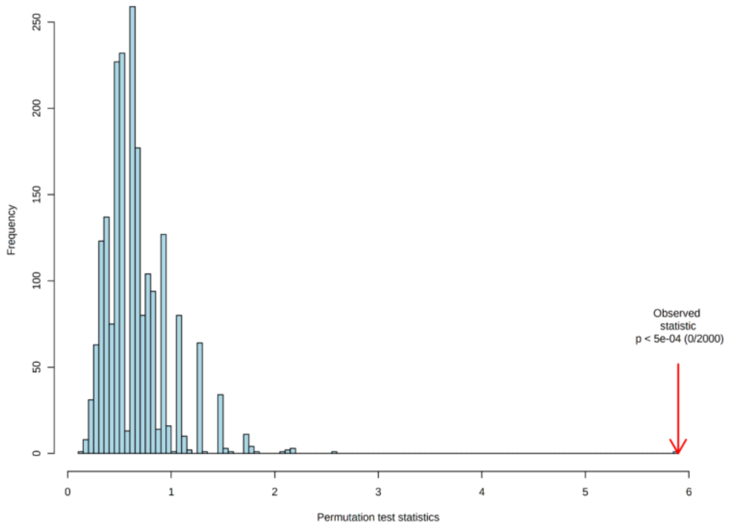

(B)

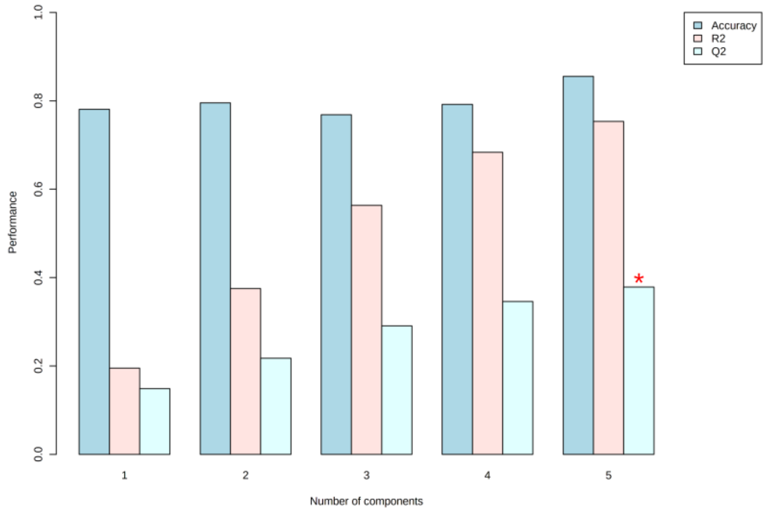

Measure	1 comps	2 comps	3 comps	4 comps	5 comps
Accuracy	0.78067	0.79533	0.76867	0.792	0.85533
R2	0.19477	0.37535	0.56367	0.68374	0.75348
Q2	0.14873	0.21761	0.2907	0.3459	0.37867

Figure S2. Validation of the statistical model used (A) permutation (2000 times) and (B) cross-validation

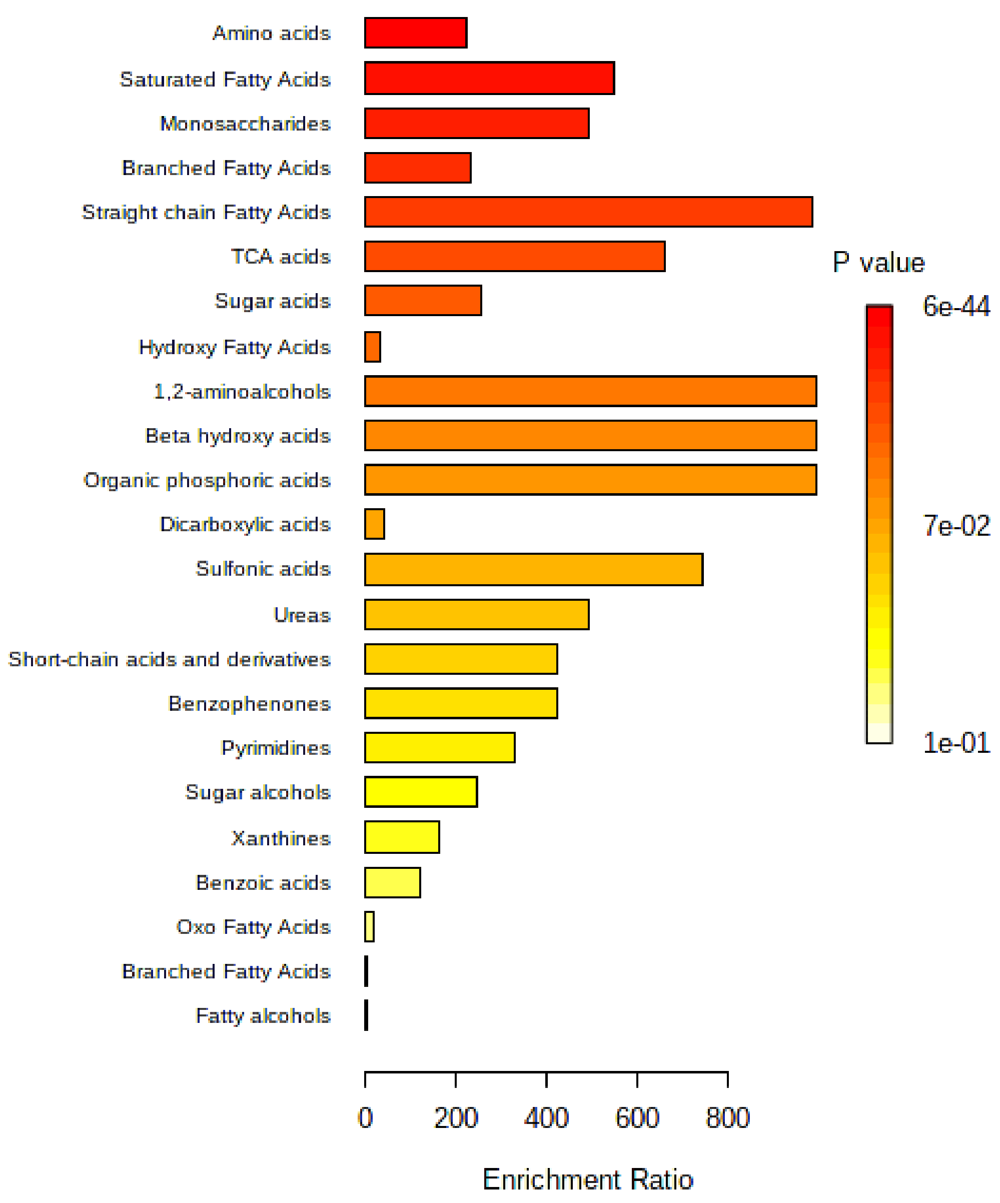

Figure S3. Metabolic pathway

Evaluation of derivatization reagent

(A)

(B)

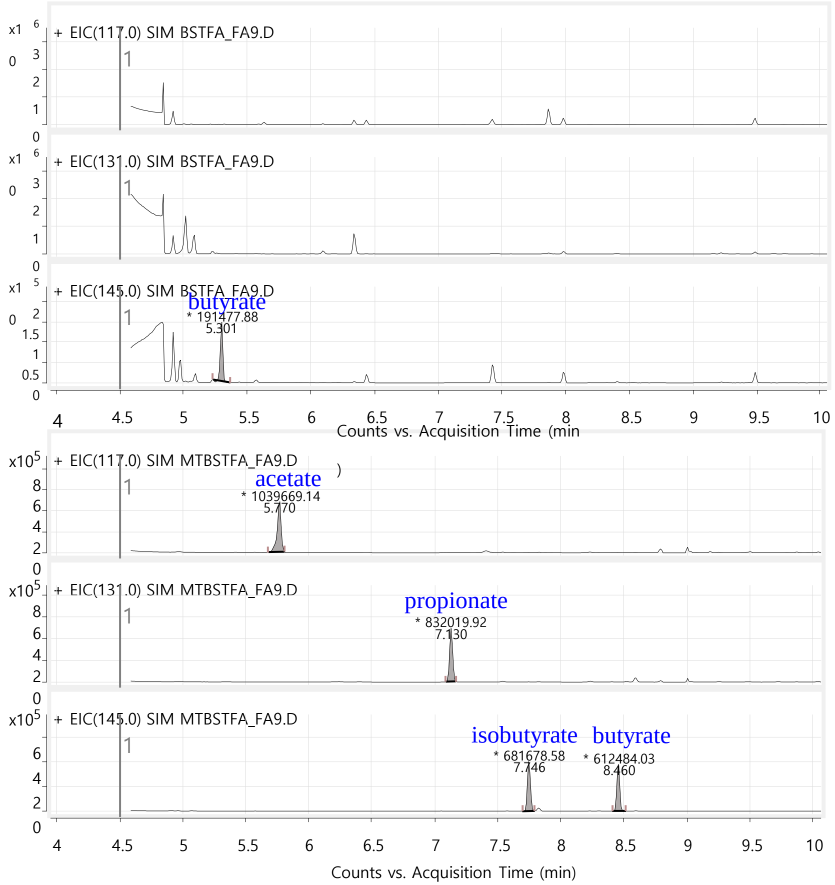

Figure S4. Comparison of derivatized-SCFA chromatograms using MTBSTFA and BSTFA reagent; (A) trimethylsilylized-SCFA; (B) *tert*-butyldimethylsilylized-SCFA; when derivatized with BSTFA, selectivity of acetate, propionate and isobutyrate were worse than MTBSTFA.

Optimization of preparation method

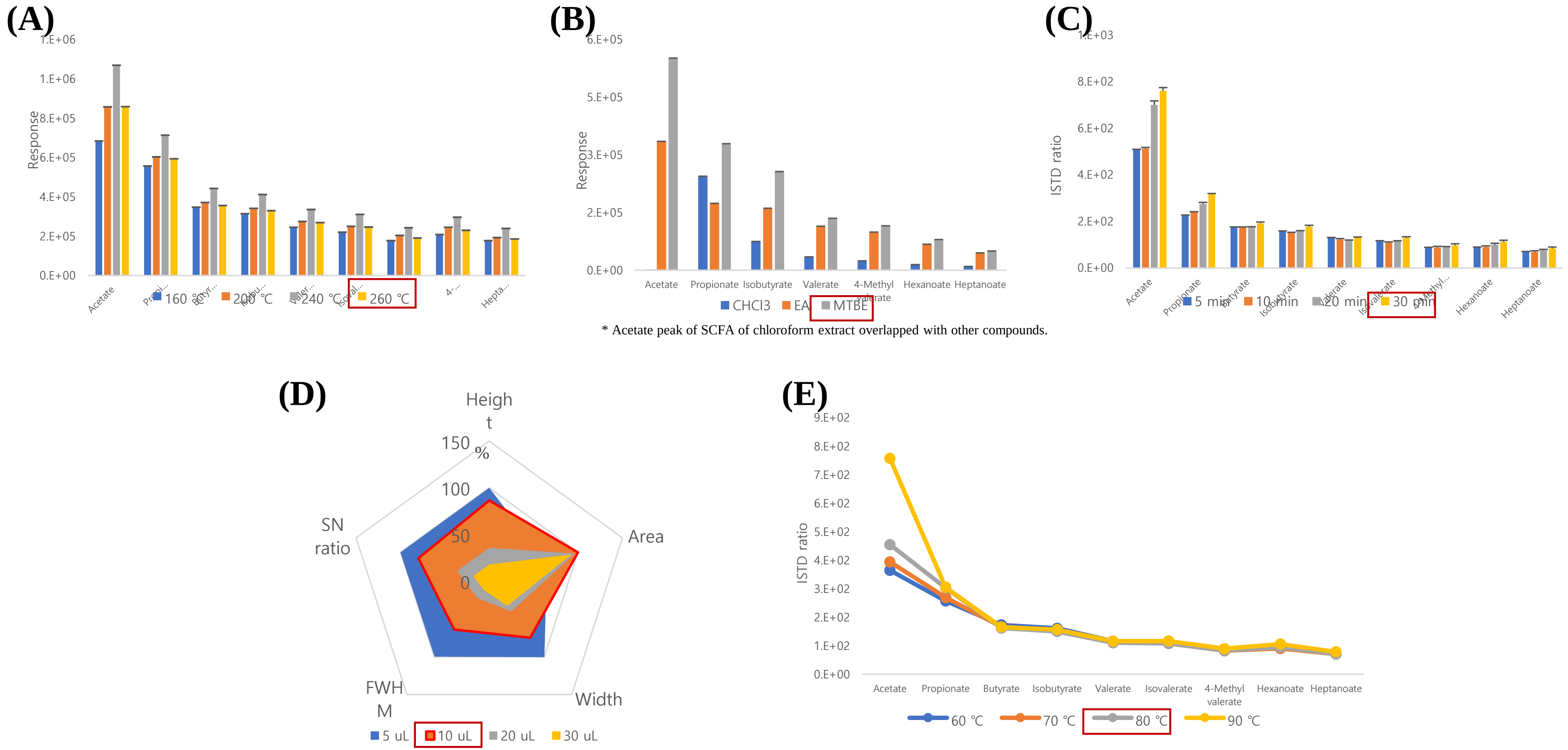

Figure S5. Optimization of preparation process of short chain fatty acid; (A) optimization of inlet temperature of GC, (B) extraction solvent screening, (C) optimization of derivatization time, (D) optimization of derivatization reagent volume, (E) optimization of derivatization temperature

Validation of analytical procedures

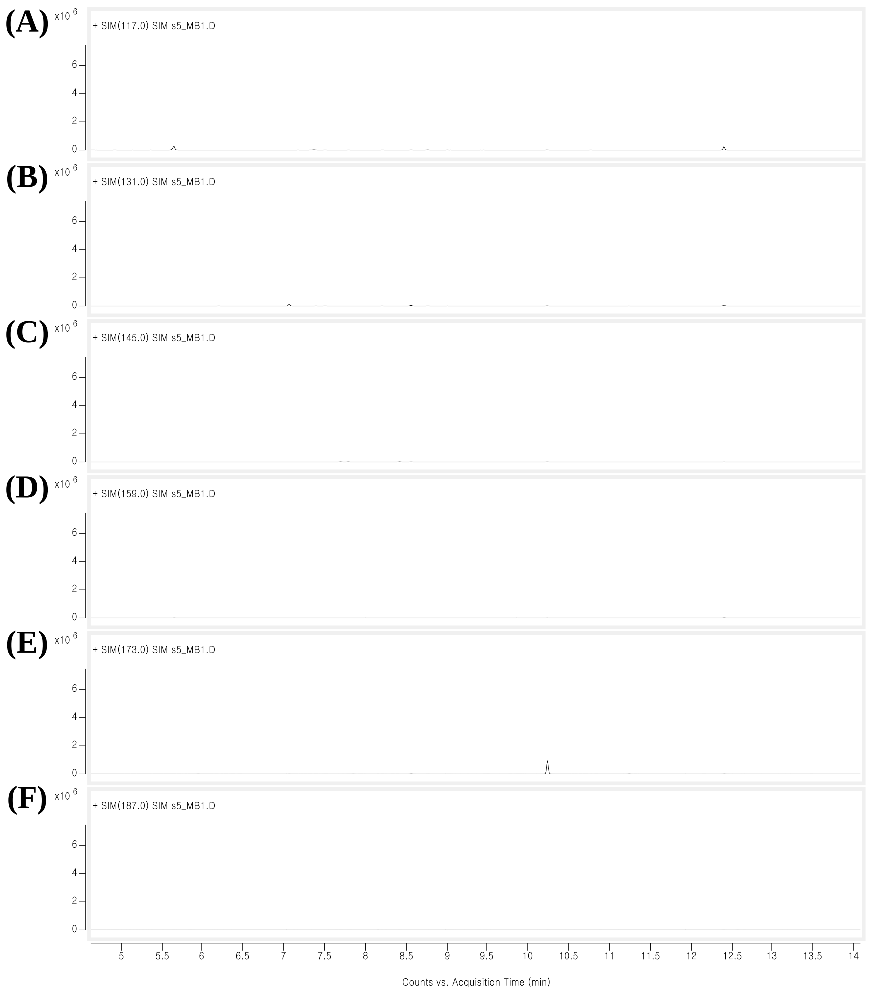

Figure S6. GC/MS chromatogram of blank sample (A) acetate, (B) propionate, (C) isobutyrate, butyrate, (D) isovalerate and valerate, (E) 4-methyl valerate and hexanoate and, (F) heptanoate

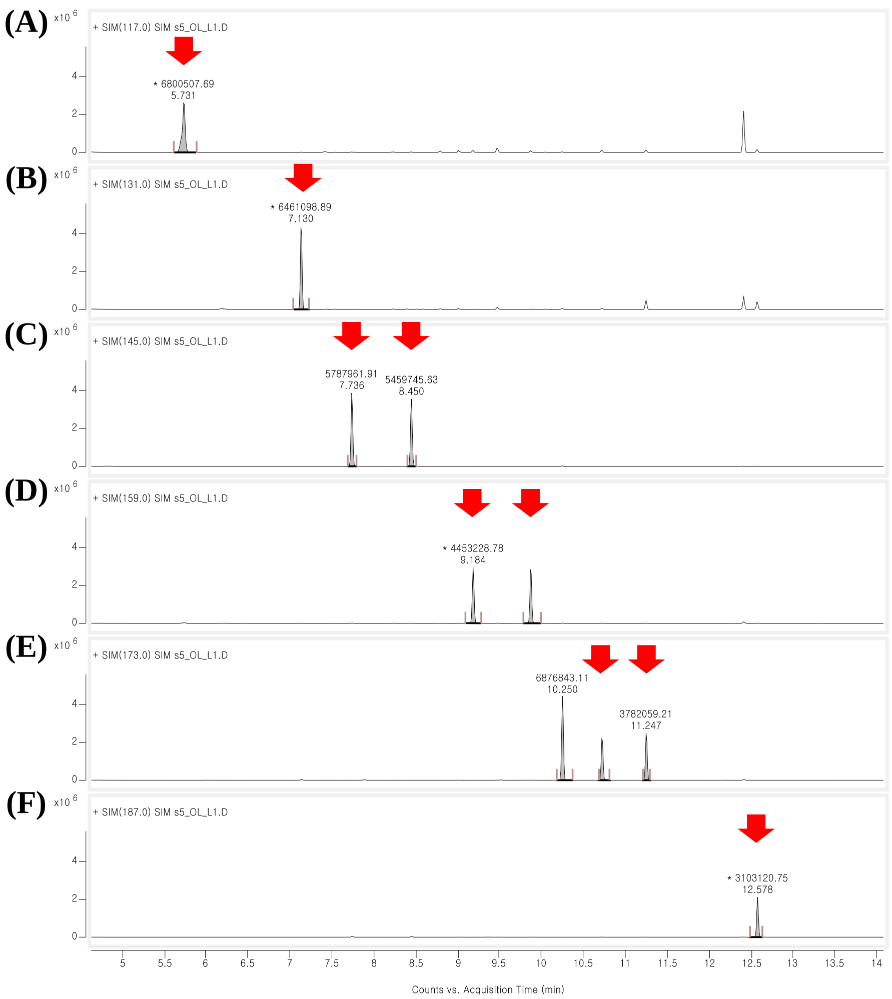

Figure S7. GC/MS chromatogram of blank sample spiked nine SCFAs (A) acetate, (B) propionate, (C) isobutyrate, butyrate, (D) isovalerate and valerate, (E) 4-methyl valerate, hexanoate, and (F) heptanoate

Validation of analytical procedures

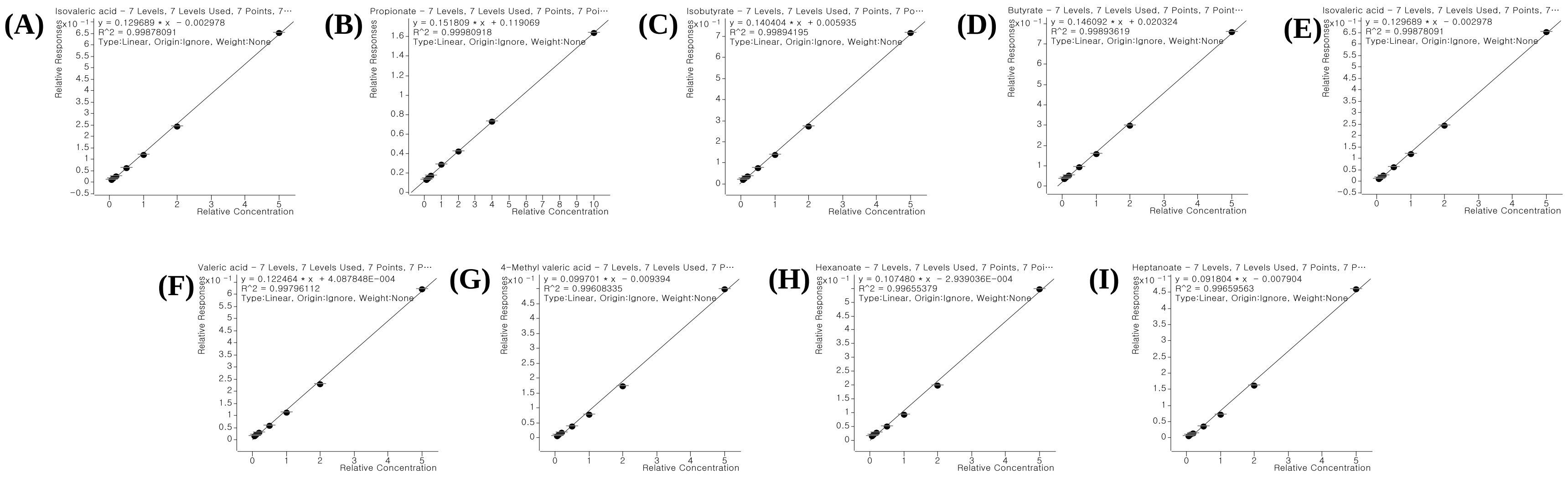

Figure S8. Calibration curves of nine SCFAs (A) acetate, (B) propionate, (C) isobutyrate, (D) butyrate, (E) isovalerate, (F) valerate, (G) 4-methyl valerate, (H) hexanoate, and (I) heptanoate

Table S4. Range of calibration curves of nine SCFAs

Target compound	Concentration range (μg/mL)		
Acetate	0.3	-	30
Propionate	0.1	-	10
Isobutyrate, butyrate, isovalerate, valerate, 4-methyl valerate, hexanoate, heptanoate	0.05	-	5

Validation of analytical procedures

Table S5. Accuracy, precision, and recovery results of nine SCFAs

Compound	Spiked conc. (ng/mL)	Intra-day (n=5)		Inter-day (n=3)		Recovery (%)	RSD (%)
		Accuracy (%)	RSD (%)	Accuracy (%)	RSD (%)		
Acetate	0.3	109.1	19.0	99.8	10.7	-	-
	0.6	101.8	13.4	98.3	18.6	84.0	8.2
	6	114.8	13.6	101.7	12.8	87.3	14.0
	30	91.9	8.8	93.5	4.2	91.6	13.4
Propionate	0.1	98.2	12.4	103.6	7.4	-	-
	0.2	94.2	8.5	100.7	7.7	93.9	13.0
	2	109.0	7.2	103.5	5.4	86.5	2.9
	10	95.1	6.8	94.5	6.5	84.9	4.8
Isobutyrate	0.05	95.2	11.3	111.4	12.9	-	-
	0.1	113.2	10.0	101.5	14.7	89.7	2.8
	1	111.1	8.4	108.1	4.6	83.7	6.2
	5	95.7	4.0	98.0	2.7	83.7	8.7
Butyrate	0.05	101.6	9.4	100.0	10.1	-	-
	0.1	93.4	11.7	89.6	7.9	89.6	3.8
	1	110.9	5.6	106.1	4.1	82.1	6.0
	5	97.2	3.7	97.0	1.9	80.1	8.9
Isovalerate	0.05	112.5	1.1	100.2	11.2	-	-
	0.1	93.1	6.9	92.0	3.1	84.6	3.4
	1	104.1	6.7	104.0	4.7	82.5	3.2
	5	100.8	2.3	100.1	1.2	82.3	7.6
Valerate	0.05	89.6	3.5	98.8	8.1	-	-
	0.1	88.9	13.0	88.7	9.7	85.3	3.7
	1	101.0	5.5	104.2	5.9	80.4	5.0
	5	102.0	1.4	102.4	3.4	79.0	7.9
4-Methyl valerate	0.05	85.8	3.3	102.5	13.2	-	-
	0.1	99.4	8.8	95.3	11.5	82.2	1.7
	1	100.4	8.2	102.6	10.3	79.5	6.5
	5	102.5	1.4	104.6	5.6	76.7	5.7
Hexanoate	0.05	83.8	14.2	101.1	14.9	-	-
	0.1	95.4	7.1	92.0	4.5	91.2	9.2
	1	97.4	3.4	101.8	7.0	79.8	6.8
	5	104.4	1.6	104.5	4.2	80.7	12.8
Heptanoate	0.05	84.8	10.2	92.3	11.8	-	-
	0.1	93.0	11.6	92.8	9.6	81.8	4.4
	1	93.3	5.3	100.7	10.7	78.6	5.3
	5	104.7	3.0	104.6	3.9	75.7	8.5

Study population

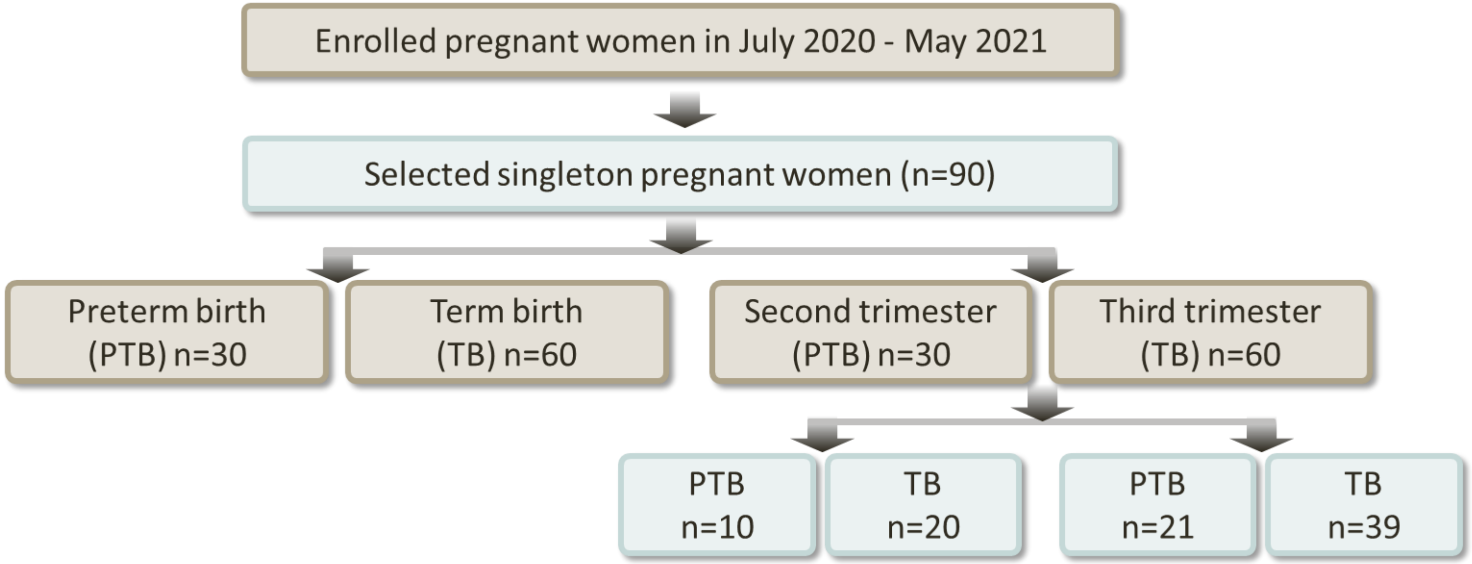

Figure S9. Study flowchart for subject selection criteria

Study population

Table S6. Clinical characteristics of subjects

characteristics	PTB (n=30)	TB (n=60)	P value
Age (years)	31.9±4.1	33.1±3.9	0.19
Pre-pregnancy BMI (kg/m2)	22.3±3.0	21.4±2.5	0.14
GAS (weeks)	29.8±5.7	31.3±7.4	0.29
GAB (weeks)	33.0±3.7	38.7±0.9	<0.001

Data are presented as Mean±SD, $p<0.05$ considered as significant. PTB: Preterm birth; TB: Term birth; BMI: Body mass index; GAS: gestational age at sampling; GAB: gestation age at birth
